# The transcriptional regulator VAL1 promotes Arabidopsis flowering by repressing the organ boundary genes *BOP1* and *BOP2*

**DOI:** 10.1093/plphys/kiaf160

**Published:** 2025-04-24

**Authors:** Yajiao Cheng, Benjamin J M Tremblay, Vicente Balanzà, Alvaro S Larran, Julia I Qüesta

**Affiliations:** Centre for Research in Agricultural Genomics (CRAG), CSIC-IRTA-UAB-UB, Campus UAB, Bellaterra, Barcelona 08193, Spain; Centre for Research in Agricultural Genomics (CRAG), CSIC-IRTA-UAB-UB, Campus UAB, Bellaterra, Barcelona 08193, Spain; Instituto de Biología Molecular y Celular de Plantas (IBMCP), Consejo Superior de Investigaciones Científicas—Universitat Politècnica de València (CSIC-UPV), Valencia 46022, Spain; Centre for Research in Agricultural Genomics (CRAG), CSIC-IRTA-UAB-UB, Campus UAB, Bellaterra, Barcelona 08193, Spain; Centre for Research in Agricultural Genomics (CRAG), CSIC-IRTA-UAB-UB, Campus UAB, Bellaterra, Barcelona 08193, Spain

## Abstract

The transition to reproductive development is a critical step in the plant lifecycle and relies on the integration of intrinsic and environmental signals. Several different pathways controlling flowering time function downstream of the perception of environmental cues such as day length (photoperiodic pathway) and seasonal temperature (vernalization and ambient temperature pathways). In addition, the phytohormone gibberellin (GA) induces the floral transition under noninductive photoperiod. In the model plant Arabidopsis (*Arabidopsis thaliana*), the transcriptional repressor VIVIPAROUS1/ABSCISIC ACID INSENTIVE3 (ABI3)-LIKE1 (VAL1) triggers the stable repression of the floral repressor *FLOWERING LOCUS C* (*FLC*) during vernalization. However, the involvement of VAL1 in other flowering pathways remains unclear. In this work, we combined genetic and transcriptomic approaches to investigate the requirement of VAL1 for flowering activation under different day lengths. We found that VAL1, but not its sister protein VAL2, is required to induce the floral transition both under long and short days. The delayed flowering time of *val1* mutant plants was fully bypassed by exogenous GA application. We demonstrated that VAL1-mediated induction of flowering occurs partially via the direct epigenetic repression of the organ boundary genes *BLADE-ON-PETIOLE1* (*BOP1*) and *BOP2*. Our work thus expands the repertoire of VAL target genes and further demonstrates the pleiotropic role of VAL factors in regulating Arabidopsis development.

## Teaser

The transcriptional repressor VAL1 induces Arabidopsis flowering time by restricting the expression of the organ boundary genes *BOP1* and *BOP2* both under long- and short-day photoperiods.

## Introduction

Flowering time control in plants is highly responsive to environmental cues. Perception of light and temperature is key to align flowering to the correct season to maximize reproductive success. In the temperate plant Arabidopsis (*Arabidopsis thaliana*), different flowering pathways have been defined that rely on registration of environmental signals over time. In winter annual Arabidopsis ecotypes, prolonged exposure to winter cold temperature, a process known as vernalization, is interpreted as a flowering-promoting signal and flowering occurs rapidly in the following spring (Hepworth and Dean 2015). In summer annual Arabidopsis, moderate increases in ambient temperature (thermosensory pathway) can also accelerate flowering (Capovilla et al. 2015; Jin and Ahn 2021). In parallel to seasonal temperature fluctuations, day length (photoperiod) changes gradually throughout the year. Activation of the Arabidopsis photoperiodic flowering pathway (long days, LD) leads to transcriptional activation of *FLOWERING LOCUS T* (*FT*), which takes place in leaf tissues under long days (Andrés and Coupland 2012; Song et al. 2015; He et al. 2020). In fact, both photoperiodic and temperature-dependent flowering pathways converge in *FT* activation. Mobilization of FT protein from leaves to the shoot apical meristem (SAM) is required for floral initiation. At the shoot apex, FT forms a complex with the basic-leucine zipper (bZIP) transcription factor FD (Abe et al. 2005; Wigge et al. 2005; Jaeger et al. 2013). Functional assembly of the FT–FD complex is essential for the induction of inflorescence meristem (IM) identity genes *SUPPRESSOR OF CONSTANS1 (SOC1)*, *AGAMOUS-LIKE24 (AGL24)*, and *FRUITFULL (FUL)*, which in turn promote activation of floral meristem (FM) identity genes *LEAFY (LFY)*, *APETALA1 (AP1)*, and *CAULIFLOWER (CAL)* (Wigge et al. 2005; Collani et al. 2019) thus conferring floral fate.

Promotion of flowering through vernalization relies on the epigenetic silencing of the major Arabidopsis floral repressor gene *FLOWERING LOCUS C* (*FLC*). FLC blocks flowering by direct repression of *FT* (Searle et al. 2006). Increasing weeks of winter cold induce gradual and quantitative downregulation of *FLC* expression, until it reaches very low levels (Berry and Dean 2015). The maintenance of *FLC* repression during spring, achieved by the vernalization-specific PLANT HOMEODOMAIN (PHD) proteins—POLYCOMB REPRESSIVE COMPLEX 2 (PHD–PRC2), releases *FT* expression to promote the floral transition. Epigenetic silencing of *FLC* is triggered by the sequence-specific DNA-binding protein VIVIPAROUS1/ABI3-LIKE1 (VAL1) (Qüesta et al. 2016; Yuan et al. 2016), which binds to an *FLC* intragenic site during cold, facilitating the recruitment of PHD–PRC2 to the locus. Following VAL1/PHD–PRC2 binding, *FLC* chromatin becomes enriched with H3K27me3 and the gene remains transcriptionally inactive. Due to its ability to guide PHD–PRC2 to *FLC* chromatin during cold, VAL1 could be considered a component of the vernalization genetic pathway. However, analysis of mutant combinations of *VAL1* and *PHD–PRC2* including *VERNALIZATION INSENSITIVE3 (VIN3)* and *VERNALIZATION2* (*VRN2)* have demonstrated a synergistic effect on flowering time (Qüesta et al. 2016). Following vernalization, the *val1 vin3* and *val1 vrn2* double mutant plants flowered substantially later than the single mutants *val1*, *vin3*, and *vrn2*. In fact, *val1 vin3* and *val1 vrn2* were unable to flower even after a 20-wk-long vernalization treatment. These observations suggest that, in addition to vernalization, VAL proteins may influence other flowering pathways in Arabidopsis.

Besides environmental signals, endogenous cues such as the phytohormone gibberellic acid (GA), plant age and carbohydrate status are key factors that promote flowering (Andrés and Coupland 2012; Conti 2017; Izawa 2021). Mutants defective in GA biosynthesis display moderate late flowering under long-day conditions, but nonflowering phenotypes under short-day conditions (Wilson et al. 1992), indicating the obligate requirement for GA when the photoperiod is disrupted. Application of exogenous GA can overcome the delay in flowering under short day (SD) (Langridge 1957). GA signaling is largely mediated by a class of nuclear proteins, globally referred to as DELLA, which act as negative regulators of GA signaling (Harberd 2003). There are 5 DELLA genes in Arabidopsis, with both specific and redundant functions (Davière and Achard 2013). GA binding to the GIBBERELLIN INSENSITIVE DWARF1 (GID1) receptor promotes proteasome-mediated degradation of DELLA proteins (Silverstone et al. 2001; Willige et al. 2007; Murase et al. 2008).

Floral induction under SD conditions also requires the age-dependent regulation of the levels of 2 microRNAs, miR156 and miR172 (Kinoshita and Richter 2020; Izawa 2021). The highly abundant miR156 is expressed in leaves during juvenile growth (Axtell and Bartel 2005; Schwab et al. 2005). To confer a gradual transition from the juvenile-to-adult phase, miR156 progressively declines in successively developing shoot-derived leaf primordia (He et al. 2018). miR156 reduction results in an increase of miR156-targeted *SQUAMOSA-PROMOTER BINDING PROTEIN-LIKE* (*SPL*) genes both in leaves and the shoot apex (Wang et al. 2009; Yamaguchi et al. 2009). Subsequently, *SPL* activates the transcription of miR172 loci, which in turn silences a family of *AP2* transcriptional repressors of *FT*. Thus, reduction of miR156 results in increased levels of miR172 leading to *FT* activation and flowering (Wu et al. 2009), although more recent work has shown that miR172 may function independently of miR156 (Zhao et al. 2023). Transcription of *MIR156* is repressed at the adult phase by the epigenetic function of PRC1 and PRC2 (Picó et al. 2015; Xu et al. 2016a; Xu et al. 2016b), which is likely dependent on the function of VAL1 protein. Reduced VAL1 activity significantly delays the timing of vegetative phase change through both miR156-dependent and independent mechanisms (Fouracre et al. 2021). *val1* mutant delay in reaching the adult phase may affect their timing to acquire competence to flower, critical for plants growing under noninductive photoperiods.

Acquisition of FM and the maintenance of proper meristem architecture are important to regulate the floral transition. In plant meristems, lateral organ boundaries separate the meristematic zone containing undifferentiated cells from the lateral organs containing differentiated cells. At the shoot apex, *BLADE-ON-PETIOLE1* (*BOP1*) and *BOP2* genes, which encode 2 BTB (for Broad-complex, Tramtrack, Bricà-brac)-ankyrin transcriptional coactivators, are specifically expressed at the base of lateral organs, adjacent to the boundary (Ha et al. 2004; Hepworth et al. 2005; Norberg et al. 2005). *BOP* genes function in the determination of leaf, flower, inflorescence, and root nodule architecture (Ha et al. 2004, 2007; Hepworth et al. 2005; Norberg et al. 2005; Karim et al. 2009; Xu et al. 2010; Couzigou et al. 2013; Khan et al. 2012, 2015, p. 20). Ectopic expression of *BOP* genes outside the lateral organ boundaries, expanding into the meristematic zone, delays the floral transition in Arabidopsis (Andrés et al. 2015), likely due to the capacity of *BOP1* and *BOP2* to repress *FD* transcription at the shoot apex. Thus, highly controlled spatial and cell-specific regulation of organ boundary genes at the shoot meristem ensures the floral transition.

In this work, we explored the flowering behavior of *val* mutants under different photoperiod regimes. We found that VAL1 is required to induce flowering under different day length conditions. Through transcriptomic and genetic analyses, we have identified the organ boundary genes *BOP1* and *BOP2* as downstream targets directly repressed by VAL1 to promote the Arabidopsis floral transition. Our work contributes to expanding the gene networks regulated by VAL proteins to induce Arabidopsis developmental transitions.

## Results

### VAL1 protein is required for Arabidopsis flowering both under inductive and noninductive photoperiod

**Figure 1.**
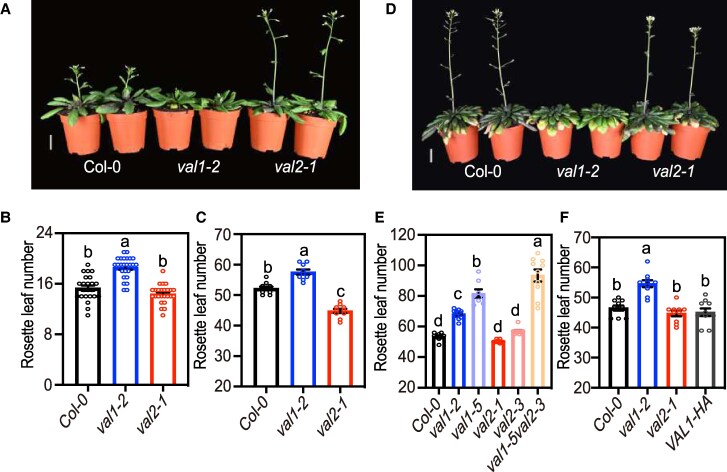
VAL1 function is required to induce flowering under inductive and noninductive photoperiod. **A)** and **D)** Flowering phenotype of 30-d-old Col-0 (WT), *val1* and *val2* plants grown under long-day photoperiod (**A**; LD, 16 h light/8 h dark) and of 73-d-old plants grown under short-day photoperiod (**D**; SD, 8 h light/16 h dark). Scale bar: 2 cm. **B)** and **C)** Rosette leaf number at bolting of WT, *val1* and *val2* plants grown under either LD **(B)** or SD **(C). B)** and **C)**, *n* ≥ 10. **E)** Rosette leaf number of WT, *val1*, *val1-5*, *val2*, *val2-3* and *val1-5 val2-3* double mutants grown under SD (*n* = 10). **F)** Rosette leaf number of WT, *val1*, *val2*, and *VAL1* complementation lines (*pVAL1:VAL1-3xHA-62*) grown under SD (*n* = 10). Values in **B)** to **C)** and **E)** to **F)** are means ± SEM. Different letters indicate significant differences as determined using Ordinary one-way ANOVA Multiple comparisons (*P*  *<*  *0.05*).

VAL proteins are integral players of the vernalization mechanism (Qüesta et al. 2016; Yuan et al. 2016; Mikulski et al. 2022). However, the function of these proteins in other flowering pathways remains poorly investigated. We analyzed the flowering behavior of Arabidopsis *val1-2* and *val2-1* single mutant alleles (in the text also referred to as *val1* and *val2*, respectively) growing at ambient temperature of 22 °C under LD photoperiod (LD, 16 h light, 8 h dark). We observed that *val1* plants flowered slightly but significantly later than *val2* and wild-type (WT) Col-0 plants (Fig. 1A), producing a significantly higher number of leaves at bolting (Fig. 1B). In contrast to *val1*, flowering of *val2* single mutant plants was equivalent to WT (Fig. 1B). Next, we tested flowering induction under noninductive photoperiod (SD, 8 h light, 16 h dark). When growing under SD, *val1* mutant exhibited a marked delay in flowering time (Fig. 1, C and D). Under these conditions, *val2* showed an acceleration of flowering compared with WT (Fig. 1C).

The flowering phenotypes of *val1* and *val2* were investigated in additional mutant alleles under SD (Fig. 1E). Like *val1*, *val1-5* allele also flowered later than WT under SD, whereas the *val2-3* allele flowered with equivalent number of leaves as *val2* and WT plants. In this case, we did not capture the acceleration of flowering in the *val2-1* background that we had observed in Fig. 1C. Interestingly, the *val1-5 val2-3* double mutant flowered significantly later than *val1-5* single mutants, possibly due to the contribution of the *val2-3* allele. This redundant effect of *VAL2* in a *VAL1* deficient background is not unique to the floral transition, as it has previously been described for other developmental transitions (Suzuki et al. 2007; Tsukagoshi et al. 2007; Yang et al. 2013a; Yuan et al. 2021). Finally, the late-flowering phenotype of *val1* was complemented with a *VAL1* genomic construct carrying a C-terminal translational fusion to HA tag (*pVAL1:VAL1-3xHA*; Fig. 1F and Supplementary Fig. S1, A to C). Four independent *VAL1-HA* transgenic lines flowered at the same time as WT plants (Supplementary Fig. S1C). These results demonstrate a prominent role of VAL1 in the control of flowering time in Arabidopsis both under LD and SD, while VAL2 seems to only be required in the absence of functional VAL1.

### VAL1 accelerates flowering under noninductive photoperiod partially through the repression of *FLC*

In winter annual Arabidopsis, VAL1 triggers the epigenetic silencing of *FLC* during winter, counteracting the positive effect of FRIGIDA (FRI) on *FLC*, and allowing spring flowering (Qüesta et al. 2016). Delay of flowering time in *val1 FRI* mutant was associated with elevated levels of *FLC* expression before, during and after vernalization treatment. *FLC* is a main component of the vernalization pathway, and its implication in other flowering pathways remains much less studied. Thus, we sought to investigate whether the late-flowering phenotype of *val1* was due to increased *FLC* levels.

**Figure 2.**
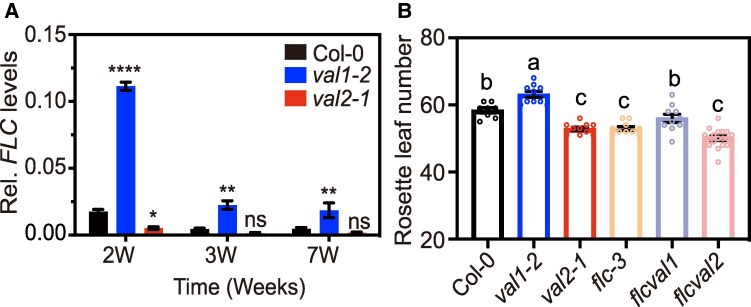
VAL1 accelerates flowering in SD partially through the transcriptional repression of *FLC*. **A)** Relative (Rel.) *FLC* transcript abundance tested by RT-qPCR in Col-0 (WT), *val1* and *val2* mutant plants grown under SD. Seeds were stratified during 72 h in the dark at 4 °C, and then transferred to the light for germination at 22 °C in SD. Plants were incubated during 2, 3, and 7 wk before sample collection. Leaf material was collected for total RNA isolation. Values are means ± SEM (*n* ≥ 3). *ACTIN2* was used as normalization control. Different stars indicate significant differences as determined using Ordinary two-way ANOVA Multiple comparisons (*P  *<*  *0.05*, **P  *<*  *0.01*, ****P  *<*  *0.0001*; ns, not significant). **B)** Flowering time of WT, *val1*, *val2* and *flc-3* single mutants, and *flc val1* and *flc val2* double mutant plants grown under SD, measured as rosette leaf number. Values are means ± SEM (*n* ≥ 8). Different letters indicate significant differences as determined using Ordinary one-way ANOVA Multiple comparisons (*P*  *<*  *0.05*).

Under LD conditions, we observed that *FLC* expression was higher in *val1* compared with *val2* and WT (Supplementary Fig. S2A). These results agreed with previous transcriptomics analyses that have reported increased *FLC* mRNA levels in the *val1 val2* double mutants (Suzuki et al. 2007; Yuan et al. 2021). In addition, a recent report has shown that disruption of *FLC* expression in *val1* mutant background (the authors generated *val1-2 flc-6* double mutant plants) could suppress the late-flowering phenotype of *val1* mutant in LD conditions (Jing et al. 2019). The higher *FLC* levels of *val1* mutant are complemented with the *VAL1-HA* genomic construct (Supplementary Fig. S1B). Together, these analyses imply that VAL1 controls LD flowering via the repression of *FLC* gene. Under SD conditions, we also detected higher *FLC* mRNA levels in *val1* mutants compared with WT and *val2* (Fig. 2A). Despite the higher initial *FLC* expression levels, reduction of *FLC* in increasing weeks of growth was not impaired in *val1* (Fig. 2A and Supplementary Fig. S2A). This observation suggests that VAL1 repressive function modulates the amount of *FLC* transcripts accumulated in young Arabidopsis seedlings, while other factors may be responsible for *FLC* downregulation as growth progresses.

The consistently higher *FLC* levels in the *val1* single mutant suggested that the delay in flowering time was caused by the major effect of *FLC*. To test this possibility, we generated *val1 flc-3* double mutants and scored flowering time under SD. Deficiency in *FLC* accelerates flowering in SD compared with WT plants as previously reported (Proveniers et al. 2007). However, mutation of *FLC* did not fully suppress the late-flowering phenotype of *val1* (Fig. 2B and Supplementary Fig. S2B). The intermediate rosette leaf number at flowering of *val1 flc-3* compared with *flc-3* and *val1* single mutants under SD suggests that VAL1 accelerates flowering partially through the transcriptional repression of *FLC* gene. This observation also implies that other VAL1 downstream targets may be involved in the regulation of flowering in SD.

### GA suppresses the late-flowering phenotype of *val1* mutant

We next sought to investigate the mechanism whereby VAL1 facilitates flowering under noninductive SD conditions. Floral induction in SDs requires the activity of the phytohormone GA and the age-dependent reduction in the levels of miR156, which is one of the most abundant miRNAs in Arabidopsis with the highest levels at the seedling stage (Wilson et al. 1992; Axtell and Bartel 2005; Schwab et al. 2005). VAL1 inactivates the miR156 pathway to promote vegetative phase change (Fouracre et al. 2021) and possibly also the floral transition. However, the link between VAL1 and GA had not been investigated yet.

**Figure 3.**
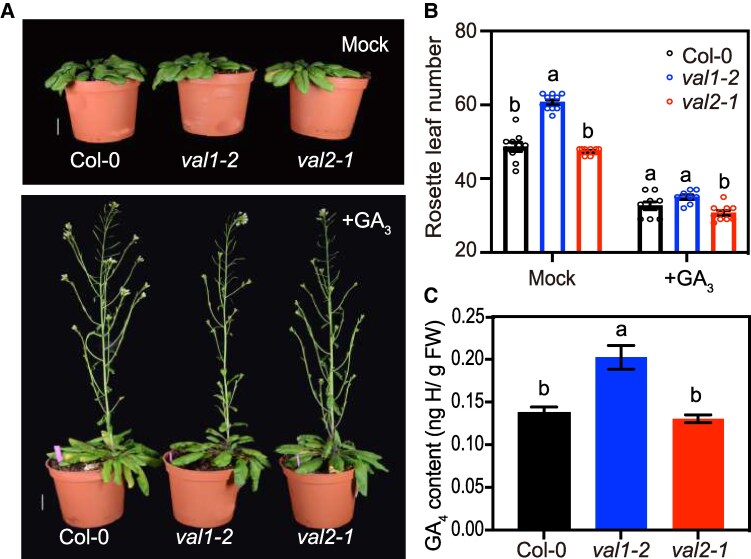
Exogenous supplementation of gibberellin (GA) overcomes the late-flowering phenotype of *val1* under SDs. **A)** Representative images of 55-d-old Col-0 (WT), *val1* and *val2* plants grown under SDs with (+GA_3_, bottom panel) or without (Mock, top panel) the supplementation of GA_3_. +GA_3_ plants were treated with 100 *μ*M GA_3_ plus 0.02% Silwet-77 twice per week, whereas Mock plants were treated with 1% ethanol plus 0.02% Silwet-77. Scale bar: 2 cm. **B)** Rosette leaf number at flowering measured under SDs. Values are means ± SEM (*n* ≥ 9). Different letters indicate significant differences as determined using Ordinary two-way ANOVA Multiple comparisons (*P*  *<*  *0.05*). **C)** Endogenous GA_4_ content was measured in the SAM of 6-wk-old plants under SDs. Values are means ± SEM of three biological replicates. *y* axis represents hormone (H) content per gram of fresh weight (FW; ng H/g FW). Different letters indicate significant differences as determined using Ordinary one-way ANOVA Multiple comparisons (*P*  *<*  *0.05*).

GAs positively regulates flowering time in Arabidopsis, particularly under SD (Wilson et al. 1992; Porri et al. 2012). To test a possible link between VAL1 and GA pathway, we analyzed the response of *val1* and *val2* mutants to exogenous GA_3_ treatment (Fig. 3). Consistent with previous reports, exogenous GA_3_ treatment accelerated flowering in WT plants grown in SD (Fig. 3, A and B). Surprisingly, exogenous GA_3_ fully suppressed *val1* delayed flowering phenotype (Fig. 3B and Supplementary Fig. S3). Under mock treatment, *val1* flowered later than WT and *val2*. However, after the application of GA_3_ all 3 genotypes flowered at equivalent time (Fig. 3, A and B and Supplementary Fig. S3).

Previous results have shown that *FLC* negatively regulates GA biosynthesis and signaling by forming a complex with its partner repressor *SHORT VEGETATIVE PHASE* (*SVP*) (Mateos et al. 2015). We reasoned that the higher *FLC* mRNA expression observed in *val1* (Fig. 2A) could lead to a reduction of endogenous, bioactive GA levels, resulting in late-flowering phenotype under SD. Gibberellins accumulate in the shoot apex as the plant ages (Wilson et al. 1992). Based on this, we tested accumulation of endogenous bioactive GA_4_ in the SAM of 6-wk-old plants. Unexpectedly, *val1* accumulated significantly higher levels of GA_4_ compared with WT and *val2* (Fig. 3C). These data suggest that the late-flowering phenotype of *val1* under SD is not the consequence of decreased levels of endogenous GAs at the SAM. Nevertheless, exogenous application of GA_3_ fully bypasses the impairment in flowering of the *val1* mutant background.

### Transcriptional responses in shoot apices of *val* mutant plants grown in SD

**Figure 4.**
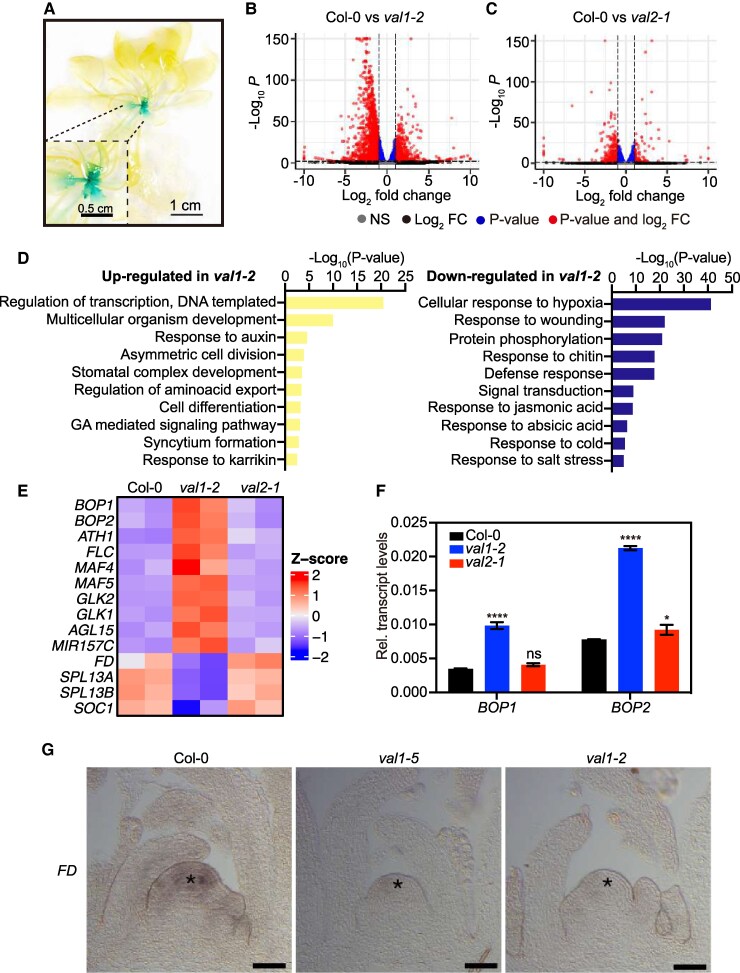
VAL1 deficiency causes misregulation of flowering genes in the Arabidopsis shoot apex. **A)** Expression of *pVAL1:VAL1-GUS* in 6-wk-old seedlings grown under SD. The inset shows magnification of the shoot area. Scale bar: 1 cm. **B)** and **C)** Volcano map of number of DEGs is upregulated in *val1*  **(B)** and *val2*  **(C)** compared to Col-0 (WT). Dashed lines in **B)** and **C)** show the FC = ±2 threshold. FC, log2 fold change; FDR, false discovery rate. **D)** GO analysis of upregulated (left, *n* = 938) and downregulated (right, *n* = 1674) genes in *val1* mutant. Top 10 representative terms are listed and ranked by *P*-value. **E)** Heat-map of upregulated and downregulated genes expression in WT, *val1* and *val2* mutant by RNA-Seq. *Z*-scores of the Transcripts Per Million (TPM) values for all samples are represented. **F)** Transcription level of *BOP1* and *BOP2* in WT, *val1* and *val2* by RT-qPCR in 6-wk-old shoot apical meristems (SAM) under SDs. Values are means ± SEM (*n* = 3). Different stars indicate significant differences as determined using Ordinary two-way ANOVA Multiple comparisons (*P  *<*  *0.05*, ****P  *<*  *0.0001*; ns, not significant). **G)**  *FD* mRNA expression in WT, *val1-5* and *val1* at 15-d-old seedlings grown under LDs by in situ hybridization. Stars indicate the SAM. Scale bar: 50 *µ*m.

To further investigate how VAL1 accelerates flowering under SD we opted for genome-wide transcriptomic analysis. Previous works have reported transcriptomic studies conducted in seedlings of single and double mutants of *VAL1* and *VAL2* grown under LD conditions (Suzuki et al. 2007; Yuan et al. 2021). However, in a recent report using a VAL1-GLUCURONIDASE (GUS) translational fusion (*pVAL1:VAL1-GUS*), Fouracre et al. (2021) demonstrated that VAL1 protein predominantly accumulates in the shoot apex of adult plants. While during embryogenesis VAL1-GUS is detected in the root and shoot apical meristems as well as in the provasculature, following germination VAL1 becomes restricted to the shoot apex and developing leaf primordia. Importantly, quantitative analysis of GUS signal demonstrated that VAL1 accumulates more strongly in the shoot apex than in leaf primordia in adult plants (Fouracre et al. 2021). Based on these observations, we wondered whether the regulation of the floral transition under SD relies on a specific function of VAL1 in the shoot apex. To tackle this question, we requested the *pVAL1:VAL1-GUS* line, and confirmed the expression pattern of VAL1-GUS in embryos (Supplementary Fig. S1D), 1-wk-old seedlings (Supplementary Fig. S1E) and 3-wk-old plants (Supplementary Fig. S1F). Furthermore, our analysis demonstrated that VAL1-GUS protein accumulates in the shoot apex and developing leaf primordia of 6-wk-old plants growing under SD (Fig. 4A), ∼10 d before bolting is detected in WT plants (Fig. 1C).

Considering the VAL1 expression pattern, we performed RNA-seq experiments to compare the transcriptomes of 6-wk-old shoot apices of *val1*, *val2* and WT plants under SD conditions (Supplementary Fig. S4 and Data Set 1). Overall, the number of differentially expressed genes (DEGs; comparing mutants and WT) was higher in *val1* (3,220 DEGs) compared with *val2* (645 DEGs; Fig. 4, B and C and Supplementary Fig. S4B, Data Set S1). A total of 1,126 genes were upregulated and 2,094 genes were downregulated in *val1*, whereas only 170 and 475 genes were up- and down-regulated in *val2*, respectively (Supplementary Fig. S4B). When comparing the genes uniquely differentially expressed in *val1*, it is interesting that the number of downregulated genes (1,827 genes) almost double the amount of upregulated genes (1,028 genes; Supplementary Fig. S4B and Data Set 2).

Gene ontology analysis was performed for *val1* up- and downregulated genes separately (Fig. 4D and Supplementary Table S1, Data Sets 3 and 4). Regulation of transcription was by far the most enriched gene ontology (GO)-term for *val1* upregulated genes followed by the regulation of different developmental processes (Fig. 4D and Supplementary Data Set 3). GA-mediated signaling pathway appeared among the top nonredundant categories for *val1* upregulated genes (Fig. 4D and Supplementary Data Set 3). Accordingly, we detected increased expression of the *DELLA* gene *GIBBERELLIN INSENSITIVE* (*GAI*) which we confirmed by Reverse transcription quantitative PCR (RT-qPCR) (Supplementary Fig. S5C). Consistent with the increased levels of GA_4_ in *val1* apices (Fig. 3C), we also detected induced accumulation of transcripts of genes involved in GA biosynthesis as well as a reduction of GA catabolic genes (Supplementary Fig. S5, A and B). The *val1* downregulated genes were overrepresented by GO-terms related to biotic and abiotic stress responses (Fig. 4D and Supplementary Data Set 4). In summary, knockdown of *VAL* genes causes transcriptomic alterations in Arabidopsis SAM of 6-wk-old plants, which are more pronounced in the *val1* mutant compared to *val2*. GO analysis supports the role of VAL1 as a transcriptional regulator of multiple genes belonging to different developmental pathways.

### VAL1 regulates flowering genes in Arabidopsis SAM

Although flowering genes did not appear enriched among the top GO-terms in the *val1* dataset (Fig. 4D and Supplementary Table S1, Data Sets 3 and 4), we were still able to identify *val1* upregulated genes that are related to reproductive development among the list of GO-term categories with lower *P*-values (Supplementary Table S2). Within these gene groups, we found 2 already characterized VAL1 targets: (i) *FLC* (Qüesta et al. 2016; Yuan et al. 2016); and (ii) *AGL15* (Chen et al. 2018) (Fig. 4E). Furthermore, we were able to identify other flowering-linked Arabidopsis genes potentially repressed by VAL1 function (Supplementary Tables S2, S3 and Fig. 4E). Among these, we observed that the *FLC* homologs *MADS AFFECTING FLOWERING* 4 (*MAF4*) and *MAF5* were upregulated in *val1* mutant. Overexpression of *MAF4* and *MAF5* led to late-flowering phenotypes, indicating that these genes also act as floral repressors (Ratcliffe et al. 2003). We also detected increased transcript levels of the *GOLDEN2 LIKE* (*GLK*) gene pair, *GLK1* and *GLK2* in *val1*. *GLK1* and *GLK2* have recently been implicated in the regulation of Arabidopsis flowering time (Susila et al. 2023). Additionally, *MIR157C* transcript expression appeared enriched in *val1* mutant, while the miR157 downstream targets *SPL13A* and *SPL13B* (Xu et al. 2016b) were downregulated (Fig. 4E).

Curiously, the organ boundary genes *BOP1* and *BOP2* were also upregulated in the shoot apex of 6-wk-old *val1* plants (Fig. 4E and Supplementary Tables S2, S3). Although more broadly recognized for their role in the determination of leaf, flower, inflorescence, and root nodule architecture (Hepworth et al. 2005; Norberg et al. 2005; Ha et al. 2007; McKim et al. 2008; Karim et al. 2009; Xu et al. 2010; Khan et al. 2012, 2015; Couzigou et al. 2013; Crick et al. 2022; Hu et al. 2023), *BOP* genes also participate in Arabidopsis floral induction. A previous study showed that ectopic activation of *BOP* genes outside their restricted expression zones in the Arabidopsis shoot apex strongly reduced *FD* accumulation (Andrés et al. 2015). Indeed, increased expression levels of *BOP1* and *BOP2* in 6-wk-old SAM of *val1* mutants grown under SD were confirmed by RT-qPCR (Fig. 4F). Accordingly, *FD* expression was downregulated in *val1* (Fig. 4, E and G and Supplementary Table S3), which correlated with the delayed flowering phenotype of *val1* plants under SD. The IM identity gene *SOC1* was downregulated in *val1* mutant (Fig. 4E and Supplementary Fig. S5D), likely as a result of impaired activation of *FD*. *ARABIDOPSIS THALIANA HOMEOBOX GENE1* (*ATH1*) also appeared upregulated in our dataset (Fig. 4E and Supplementary Table S2, Fig. S5E). *ATH1* gene has been proposed both as downstream target (Khan et al. 2015) and regulator of *BOP* genes (Ejaz et al. 2021). Interestingly, *ATH1* prevents the floral transition through the positive regulation of *FLC* gene (Proveniers et al. 2007). Thus, augmented levels of *BOP* and *ATH1* transcripts correlate with the late-flowering phenotype of *val1* mutant plants. Together, our transcriptomic analysis provided the evidence of a prominent role of VAL1 in the regulation of Arabidopsis flowering genes in the SAM under SD conditions.

### Epigenetic repression of the organ boundary genes *BOP1* and *BOP2* requires VAL1 function

**Figure 5.**
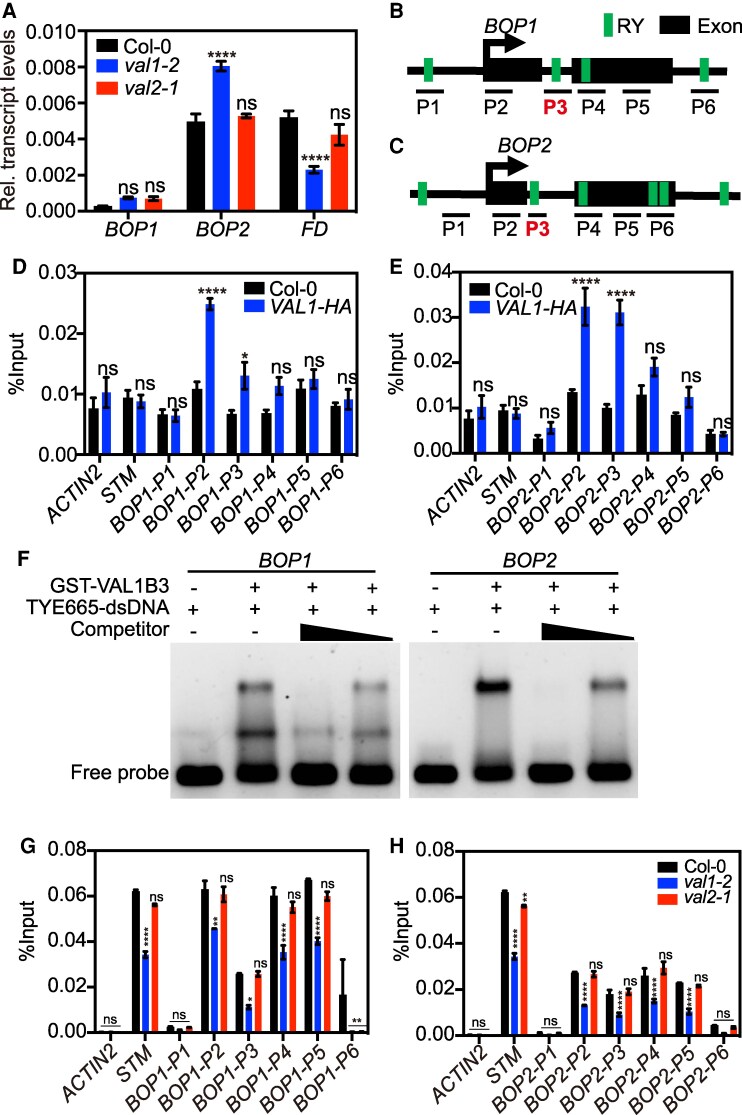
VAL1 triggers epigenetic silencing of the organ boundary genes *BOP1* and *BOP2*. **A)** Relative (Rel.) transcript levels of *BOP1*, *BOP2* and *FD* in 2-wk-old Col-0 (WT), *val1* and *val2* plants grown under LD. Transcript abundance was measured by RT-qPCR. Values are means ± SEM of three biological replicates (*n* = 9). *ACTIN2* was used as normalization control. Different stars indicate significant differences as determined using Ordinary two-way ANOVA Multiple comparisons (****P  *<*  *0.0001*; ns, not significant). **B)** and **C)** Schematic depicting *BOP1*  **(B)** and *BOP2*  **(C)** genomic loci. Exonic regions and VAL1-specific DNA motifs (RY motifs, TGCATG) are shown in the schematic. Line bars below each gene structure depict the position of the primer pairs (P1-6) used in ChIP experiments. **D)** and **E)** ChIP-qPCR analysis using anti-HA antibody reveals the binding of VAL1 to the *BOP1*  **(D)** and *BOP2*  **(E)** gene body in 2-wk-old seedlings of WT and *VAL1-HA* grown under LDs. *ACTIN2* and *STM* were included as background controls. Values are means ± SEM of 3 biological replicates (*n* = 9). Different stars indicate significant differences as determined using Ordinary two-way ANOVA Multiple comparisons (*P < 0.05, ****P < 0.0001; ns, not significant). **F)** EMSA testing GST-VAL1B3 binding to the genomic regions of *BOP1* and *BOP2*. Two fluorescently (TYE665) labeled dsDNA probes were designed: BOP1-TYE665-dsDNA and BOP2-TYE665-dsDNA. The approximate genomic locations of BOP1-TYE665-dsDNA and BOP2-TYE665-dsDNA probe sequences are depicted in **B)** and **C)**, respectively (P3, in red). Competition experiments were performed using decreasing concentration of unlabeled probes (50× and 25× molar excess). **G)** and **H)** ChIP-qPCR analysis of H3K27me3 enrichment at *BOP1*  **(G)** and *BOP2*  **(H)** loci in 2-wk-old WT, *val1* and *val2* plants grown under LD. *STM* and *ACTIN2* were used as positive and negative controls of H3K27me3 enrichment, respectively. Values are means ± SEM of the three technical replicates in one qPCR experiment. An independent biological replicate of this H3K27me3 ChIP experiment is presented in Supplementary Fig. S6, E and F. Different stars indicate significant differences as determined using Ordinary two-way ANOVA Multiple comparisons (*P < 0.05, **P < 0.01, ****P < 0.0001; ns, not significant).

Our transcriptomic analysis revealed that *BOP* genes are upregulated in the apex of *val1* plants when grown under SD, pointing to VAL1 as a potential upstream repressor of *BOPs* under these conditions. However, a previous genetic screen placed *BOP* genes downstream of *FT* in the photoperiodic flowering pathway, with ectopic expression of *BOPs* strongly reducing *FD* transcription (Andrés et al. 2015). Thus, we evaluated the expression of *BOP* genes in *val1* whole seedlings grown under LD. Our results showed significantly higher accumulation of *BOP2* mRNA in 2-wk-old *val1* seedlings compared with WT and *val2* plants grown under LD (Fig. 5A and Supplementary Fig. S6A). In the case of *BOP1*, we consistently observed slightly higher but nonsignificant expression of *BOP1* transcript levels both in *val1* and *val2* single mutants (Fig. 5A and Supplementary Fig. S6A). Yet, higher *BOP2* levels correlated with reduced accumulation of *FD* transcript (Fig. 5A) and augmented levels of its downstream target *ATH1* (Supplementary Fig. S5F), also matching the delay in flowering time of *val1* mutant under LD (Fig. 1, A and B). These data suggested that VAL1 negatively regulates transcription of *BOP2*, and possibly also *BOP1*, under inductive photoperiod. Under noninductive photoperiod, VAL1 clearly represses expression of both *BOP1* and *BOP2* mRNA (Fig. 4, E and F).

We next explored whether VAL1 directly binds to *BOP1* and *BOP2* genomic loci. VAL1 transcriptional repressor recognizes and binds specifically to Sph/RY motifs (Qüesta et al. 2016). By sequence analysis, we identified RY motifs in the genomic regions of *BOP1* and *BOP2* genes (Fig. 5, B and C). To test VAL1 binding to *BOPs*, we conducted chromatin immunoprecipitation (ChIP)-qPCR experiments using the *pVAL1:VAL1-HA* transgenic line (Fig. 1F and Supplementary Fig. S1, A to C). We detected enrichment of VAL1-HA protein over the genic regions of both *BOP1* and *BOP2* genes in 2-wk-old seedlings grown under LD (Fig. 5, D and E). More precisely, VAL1-HA protein accumulated over the first exon of *BOP* genes. In line with our ChIP-qPCR experiments, we identified *BOP1* and *BOP2* genes within the list of target genes in the VAL1 ChIP-seq data (Supplementary Fig. S6, C and D; Yuan et al. 2021). We also performed electrophoretic mobility shift assay (EMSA) using recombinant B3 DNA-binding module of VAL1 (VAL1B3) tagged with glutathione S-transferase (GST). We confirmed that GST-VAL1B3 specifically recognizes and binds the genomic sequences of *BOP1* and *BOP2* (Fig. 5F) which contain the RY motifs located in the intronic region of each gene (Fig. 5, B and C). Moreover, competition experiments using unlabeled probes further demonstrated binding specificity of GST-VAL1B3 to *BOP1* and *BOP2* genomic sequences (Fig. 5F). Free GST protein did not bind the *BOP1* and *BOP2* probes (Supplementary Fig. S6G). Thus, these data imply that VAL1 directly binds to *BOP2* chromatin to prevent its expression. Despite the nonsignificant effect on transcript accumulation in the *val1* mutant under LD (Fig. 5A and Supplementary Fig. S6A), we also detected enrichment of VAL1 protein at the *BOP1* genomic locus.

VAL1 has been associated to epigenetic silencing by PRC2 complex to promote the Arabidopsis floral transition (Qüesta et al. 2016; Yuan et al. 2016). Accordingly, a significant proportion of VAL1 targets are silenced by PRC2 (Yuan et al. 2021). We wondered whether the repression of *BOP2* as well as the mild upregulation of *BOP1* by VAL1 under LD occurred via PRC2 activity. *BOP1* and *BOP2* exhibit a very distinct expression pattern during Arabidopsis development, accumulating at organ boundaries (Xu et al. 2010). In vegetative tissues, *BOP1* and *BOP2* are expressed at low levels and their chromatin is highly enriched with H3K27me3 (Supplementary Fig. S6, C and D), the distinctive feature of PRC2 target genes. Our H3K27me3 ChIP-qPCR in 2-wk-old Col-0 seedlings confirmed that both *BOP1* and *BOP2* gene bodies are enriched with H3K27me3 (Fig. 5, G and H and Supplementary Fig. S6, E and F). In the absence of VAL1, decreased levels of H3K27me3 correlates with higher levels of *BOP2* transcript accumulation (Fig. 5H and Supplementary Fig. S6F). In contrast, *val2* mutant accumulates WT levels of H3K27me3 over *BOP2*. Interestingly, we also observed a significant reduction in H3K27me3 enrichment at the *BOP1* locus in the *val1* mutant (Fig. 5G and Supplementary Fig. S6E), although this was not reflected in a significant upregulation of the *BOP1* mRNA under LD conditions. At this point, we wondered whether the lack of *BOP1* upregulation in *val1* under LD could be due to a technical limitation of the RT-qPCR assay, such as the use of *ACTIN2* (*ACT2*) as normalization gene control. Indeed, we found that *BOP1* and *BOP2* transcript levels were significantly upregulated when using an alternative normalization gene *POLYUBIQUITIN 10* (*UBQ10*), thereby confirming that VAL1 directly represses *BOP1* and *BOP2* under LD (Supplementary Fig. S6, A and B). Together, these results suggested that in parallel to *FLC* inactivation, VAL1 controls the Arabidopsis floral transition by promoting PRC2-mediated silencing of *BOP1* and *BOP2*.

### VAL1 controls flowering partially through the repression of *BOP1* and *BOP2*

**Figure 6.**
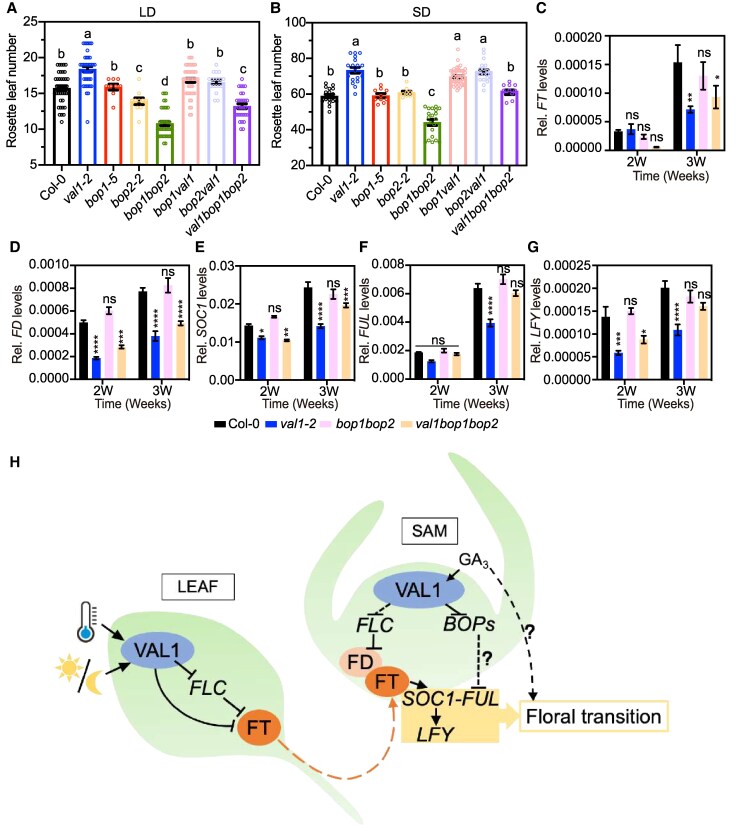
VAL1 regulates flowering time by repressing *BOP* genes. **A)** and **B)** Rosette leaf number at bolting measured for Col-0 (WT), *val1*, *bop1-5*, *bop2-2*, *bop1 bop2*, *val1 bop1*, *val1 bop2*, and *val1 bop1 bop2* plants grown in either LD (**A**, *n* ≥ 9) or SD (**B**, *n* ≥ 8). Values are means ± SEM. Different letters indicate significant differences as determined using Ordinary one-way ANOVA Multiple comparisons (*P*  *<*  *0.05*). **C)** to **G)** Relative (Rel.) transcript abundance of flowering genes *FT* (**C**), *FD*  **(D)**, *SOC1*  **(E)**, *FUL*  **(F)**, and *LFY*  **(G)** assayed by RT-qPCR. WT, *val1*, *bop1 bop2* and *val1 bop1 bop2* seeds were stratified during 72 h in the dark at 4 °C, and then transferred to the light for germination at 22 °C in LD. Plants were incubated during 2 and 3 wk before sample collection. Values are means ± SEM (*n* = 6). *ACTIN2* was used as normalization control. Different stars indicate significant differences as determined using Ordinary two-way ANOVA Multiple comparisons (*P < 0.05, **P < 0.01, ****P < 0.0001; ns, not significant). **H)** Schematic model depicting VAL1 function in the regulation of Arabidopsis flowering time. In leaves, VAL1 promotes the floral transition by promoting *FLC* downregulation in response to winter cold and both in LD and SD under standard ambient temperature conditions (22 °C), thereby releasing *FT* from FLC block. In the shoot apex, VAL1 directly represses *FLC* gene, which results in induction of *FD* mRNA. Accumulation of FD protein in the SAM forms a complex with the FT protein translocated from leaves to induce expression of IM identity genes, thus initiating reproductive development. In parallel, VAL1 also induces flowering by directly repressing *BOP1/2* in a PRC2-dependent manner, which results in the induction of *FUL* mRNA. The mechanism whereby *BOP* genes control *FUL* remains unknown (dotted line). Likewise, the role of GA in bypassing the delay flowering phenotype of *val1* mutant needs further exploration. Solid black lines indicate mechanisms either previously known or demonstrated in this work, while dotted black lines depict unknown mechanisms. Orange arrow (dotted line) depicts protein relocalization from leaf to SAM.

To confirm that VAL1 regulates flowering through the inactivation of *BOP* genes, we evaluated the genetic interaction between *VAL1* and *BOP1/2*. Despite their reported role as modulators of the *FT* signaling pathway (Andrés et al. 2015), *bop1* and *bop2* single mutants flower at equivalent time to WT plants both under LD and SD conditions (Fig. 6, A and B). Only *bop1 bop2* double mutant plants exhibited early-flowering phenotype compared with WT, confirming the redundant function of *BOP* genes in the regulation of flowering.

To test the genetic interaction of *VAL1* and *BOPs*, we conducted genetic crosses to obtain *val1 bop1/2* double and triple mutant plants and scored flowering time (Fig. 6, A and B). The loss of function of *BOP1* and *BOP2* significantly suppressed the late-flowering phenotypes of *val1* mutant both under LD (Fig. 6A) and SD (Fig. 6B). These observations supported our model that *BOP1/2* genes are downstream targets of VAL1 in the regulation of flowering. To further dissect the mechanism whereby VAL1 epigenetically represses *BOP* genes to induce the floral transition in Arabidopsis, we assessed the expression of the floral integrator genes *FT* and *FD*, as well as the IM identity (*SOC1* and *FUL*) and FM identity (*LFY*) genes, in *val1 bop1 bop2* triple mutant plants grown during 2 and 3 wk under LD photoperiod. Unexpectedly, mutation of *BOP1* and *BOP2* does not rescue the low *FT* and *FD* levels of the *val1* mutant background. In fact, *FT* and *FD* mRNA levels in *bop1 bop2* double mutant plants do not differ from those observed in WT plants (Fig. 6, C and D). Likewise, mRNA levels of the IM identity gene *SOC1* are equivalent in *val1* and *val1 bop1 bop2* plants (Fig. 6E). Accumulation of the IM identity gene *FUL* is significantly different between *val1* and *val1 bop1 bop2* (Fig. 6F). Indeed, removal of *BOP1*/*2* activity in the *val1* mutant background restored *FUL* to WT levels. These results suggest that VAL1 repression of *BOP* genes in the shoot apex may be required for the induction of *FUL* activity, independently from *FT*, *FD*, and *SOC1*. Accordingly, *LFY* mRNA levels, which are significantly low in *val1* plants, are reestablished to WT levels in *val1 bop1 bop2* plants (Fig. 6G).

Taken together, the experiments described above provide evidence that the transcriptional repressor VAL1 is required to induce the Arabidopsis floral transition under both inductive and noninductive photoperiod partially via the direct inactivation of *BOP* genes (Fig. 6H), and particularly in SD conditions, possibly also through other parallel mechanisms including *FLC* repression and GA signaling.

## Discussion

The sequence-specific DNA-binding protein VAL1 facilitates epigenetic silencing by Polycomb Repressive Complexes in Arabidopsis thereby inducing different developmental transitions including the progression from mature seed to seedling, vegetative phase change, and the switch to reproductive development. These different roles of VAL1 have been attributed to its capacity to recognize and bind to DNA, which is followed by the assembly of PRC complexes at specific target genes. In line with this, a significant number of VAL1 direct targets are marked by H3K27me3 and/or H2AK121ub at some stage during Arabidopsis development. Remarkably, several VAL1/PRC direct targets are key developmental genes (Yuan et al. 2021), many of them transcription factors that exhibit highly temporal- and cell-specific expression patterns. In the present work, we expand the list of VAL1 downstream targets by showing that *BOP1* and *BOP2* are also subject to VAL1-dependent epigenetic repression.

During the floral transition, perception of external and endogenous signals converges at the SAM to promote acquisition of IM fate (Andrés and Coupland 2012). The results described here provide evidence that the contribution of VAL1 to IM specification occurs at several nodes (Fig. 6H). In leaves, VAL1 secures epigenetic repression of *FLC*, a process also documented for flowering after vernalization (Qüesta et al. 2016; Yuan et al. 2016). These reduced levels of *FLC* due to VAL1 function under inductive LDs and warm ambient temperatures can no longer prevent *FT* activation thus the floral transition is triggered. Beyond leaf tissues, and in line with its significant accumulation at the shoot apex, we found that VAL1 directly binds and represses the organ boundary genes *BOP1* and *BOP2*. *BOP* genes had been previously implicated in the control of Arabidopsis flowering as modulators of the *FT* signaling pathway (Andrés et al. 2015). The authors of the latter work proposed that ectopic expression of *BOP* genes outside its restricted organ boundary area in the shoot apex, caused by the inactivation of the homeodomain transcription factor PENNYWISE (PNY), leads to reduced accumulation of *FD* transcripts thus delaying the floral transition. Following these previous findings, our first hypothesis was that upregulation of *BOP1* and *BOP2* in the absence of functional VAL1 would also be responsible for the reduced *FD* levels of *val1* mutant plants. However, our genetic analyses demonstrated that the induction of flowering induced by the VAL1/BOPs system occurs independently of *FD*. Instead, our results suggest that VAL1/BOPs activity converges in the activation of the IM identity gene *FUL*. Notably, the expression levels of *FUL* in *bop1 bop2* double mutant plants are indistinguishable from WT, as is also the case for *FD*, *SOC1*, and *LFY*. This lack of *FUL* and *LFY* upregulation in *bop1 bop2* (Fig. 6, F and G) aligns with prior findings by Andrés et al. (2015). In the latter work, the authors tested *LFY* expression in *bop1 bop2* mutant as well as in a *BOP1* overexpressing line (*bop1-6D*). Similar to our results, *LFY* expression levels are equivalent to WT (Col-0) in *bop1 bop2*, whereas they observed a significant decrease in *LFY* transcript levels in the *bop1-6D* plants. These data indicated that *LFY* is dramatically reduced by overexpression of *BOP1*. In line with this work, we propose that upregulation of *BOPs* in *val1* mutant leads to a reduction in *LFY* expression (Fig. 6G), which may be caused by the reduced levels of its upstream regulator *FUL* (Fig. 6F). However, it remains intriguing what causes the early-flowering phenotype of *bop1 bop2* (Fig. 6, A and B). Detailed transcriptomic analysis of the *bop1 bop2* mutant background would be required to dissect the list of *BOP* downstream target genes, likely shedding light on the role of *BOP1/2* in Arabidopsis flowering time regulation. Besides, the clearly reduced levels of *FD* transcripts in *val1* (Fig. 4G) could be the result of increased accumulation of *FLC* in apices (Fig. 4E), since this floral repressor binds directly to *FD* promoter (Searle et al. 2006).

As regulators of boundary patterning, *BOP* genes play an essential and redundant role in shaping plant architecture at different stages of development. The earliest notable defects in vegetative growth displayed by *bop1 bop2* mutant plants is the abnormal leaf morphology, including large leaves without petioles and leaflets initiated along the petiole (Hepworth et al. 2005; Norberg et al. 2005). Several elegant studies later supported the function of *BOP* genes during plant reproductive development by controlling different processes such as inflorescence architecture, FM maintenance, and floral abscission. However, despite previous analysis (Karim et al. 2009; Xu et al. 2010; Andrés et al. 2015) our knowledge on the function of *BOPs* in the regulation of the Arabidopsis floral transition remains scarce. Our work further demonstrates that *BOPs* are required to prevent the precocious switch to reproductive growth downstream of VAL1 not only under LD, but also under SD, likely via a possible repressive activity over *FUL* (Fig. 6H). The exact mechanism whereby *BOPs* could downregulate *FUL* remains unexplored. Due to their co-activator role, the prediction would be that BOP1/2 proteins are recruited by transcription factors, possibly the previously described TGACG-motif binding bZIP transcription factors (Wang et al. 2019), directly to an upstream repressor of *FUL*. Beyond the regulation of the floral transition, it remains unclear whether the other multiple *BOP1* and *BOP2* functions would also require VAL1. In principle, *val1* mutants, and even *val1 val2* double mutants, do not display the obvious *bop* mutant phenotypes in leaf shape, inflorescence architecture, flower development, and floral abscission, although these processes have not been studied in enough detail.

In addition to *BOP1/2*, our transcriptomic analysis revealed more genes implicated in Arabidopsis SD flowering that appeared miss-regulated in shoot apices of the *val1* mutant (Fig. 4E and Supplementary Table S2). Although the role of *FLC* under SD has been less studied, the *flc* mutant exhibits early-flowering phenotype (Proveniers et al. 2007; Fig. 2B and Supplementary Fig. S2B). Accordingly, our data demonstrated that VAL1 represses *FLC* expression in SDs. However, the disruption of *FLC* function does not fully suppress the *val1* late-flowering phenotype (Fig. 2B and Supplementary Fig. S2B), which is somehow expected since VAL1 has been implicated in the ageing pathway (Fouracre et al. 2021) and possibly also plays a role in the GA flowering pathway under noninductive photoperiod (Fig. 3). The *FLC* homologs *MAF4* and *MAF5* were among the *val1* upregulated genes. Interestingly, the VAL1-interacting proteins AtRING1A and AtBMI1A/B/C (Yang et al. 2013b; Qüesta et al. 2016), components of Arabidopsis PRC1, suppress the expression of *MAF4* and *MAF5* through affecting H3K27me3 levels at these loci to regulate the floral transition in *Arabidopsis* (Shen et al. 2014; Picó et al. 2015). Therefore, one possibility is that VAL1 may be mediating PRC1 and likely also PRC2 recruitment to *MAF4* and *MAF5* chromatin to trigger their epigenetic repression. However, *MAF4* and *MAF5* do not appear among the list of VAL1 direct targets according to a recent VAL1 ChIP-seq analysis conducted in 2-wk-old Arabidopsis seedlings (Yuan et al. 2021). The possibility remains that VAL1 binds to *MAF4* and *MAF5* at later stages of plant development. Thus, the direct regulation of *MAF4* and *MAF5* by VAL1 requires more detailed studies.

The metabolic status of the plant is important for the regulation of flowering time. The chloroplast produces sugars in photosynthesis and communicates with the nucleus via retrograde signaling, to control plant developmental responses (Chan et al. 2016; Hernández-Verdeja and Strand 2018). For flowering under LD, the chloroplast has a repressive function via the activity of GLK1 and GLK2 transcription factors, which upregulate the expression of *B-BOX DOMAIN PROTEIN 14/15/16* (*BBX14/15/16*) genes (Susila et al. 2023). In turn, BBXs physically interact with CONSTANS (CO) and impair CO binding to the *FT* promoter thus delaying the floral transition. The increased accumulation of *GLK1* and *GLK2* transcripts in *val1* mutants grown in SD (Fig. 4E) suggests that VAL1 may be repressing *GLKs* to induce the floral transition. Indeed, *GLK1* and *GLK2* appear as VAL1 direct targets in Arabidopsis seedlings (Yuan et al. 2021). On the other hand, as a source of sugars, the chloroplast acts as a positive regulator of flowering time (Wahl et al. 2013) through the age-dependent pathway mediated miR156 (Yu et al. 2013; Yang et al. 2013b). The application of exogenous sugars, such as sucrose, reduces the accumulation of mature miR156, a repressor of flowering time. Previous work has documented that VAL1 epigenetically silences *MIR156* (Fouracre et al. 2021), indirectly linking VAL1 with the sugar-responsive pathway. However, in our current settings (SAM of 6-wk-old plants under SD), we have not been able to detect increased levels of *MIR156* transcripts. Instead, we have observed increased expression of *MIR157* primary transcript jointly with a decreased accumulation of its downstream targets *SPL13A* and *SPL13B* (Fig. 4E). In line with our observations, recent work has shown that *MIR156/7*-regulated *SPL9* and *SPL13* control Arabidopsis leaf elongation and the juvenile-to-adult transition through the direct repression of *BOP1/2* expression (Hu et al. 2023). Although speculative, VAL1 may be restricting *BOP* genes activity via the parallel downregulation of *MIR157* transcription.

GA-mediated signaling pathway stood out as GO-term in our transcriptomic analysis. Like *val1*, mutants defective in GA biosynthesis (*ga* mutants) display moderate late flowering under LDs and a significant delay in flowering under SDs (Wilson et al. 1992), although *ga* mutants do not flower at all in SDs. Despite some similarities in flowering time shared by *val1* and *ga* mutants, the interpretation of our results regarding GA regulation in *val1* is somehow not simple. For instance, we detected higher than WT levels of bioactive GA_4_ in the apices of *val1* mutant which presented increased levels of *FLC* mRNA (Fig. 2A). These results contradict previous reports showing that high levels of *FLC* led to decrease amount of GA (Mateos et al. 2015). On the other hand, apex-specific constitutive expression of DELLA proteins has been reported to produce nonflowering phenotypes in SD conditions (Porri et al. 2012). In line with this, the absence of functional *VAL1* led to upregulation of the *DELLA* gene *GAI* (Supplementary Fig. S5C) previously linked to the regulation of the floral transition (Galvão et al. 2012), although the augmented mRNA levels may not reflect the actual levels of these DELLA proteins. While additional follow-up work will be required to fully elucidate whether VAL1 influences Arabidopsis GA responses, our results clearly demonstrated that exogenous application of GA_3_ overcomes *val1* flowering deficiency under SD conditions (Fig. 3, A and B). Therefore, the work presented here provides additional insights on the spectra of mechanisms likely controlled by the transcriptional repressor VAL1 in the regulation of Arabidopsis flowering both under inductive and noninductive photoperiods. It remains to be determined to what extent VAL1 regulates flowering in other plant species.

## Materials and methods

### Plant material and growth conditions

All plants used in this work are in the Arabidopsis (*A. thaliana*) Columbia (Col-0) background. The *val1-2* (SALK-088606) and *val2-1* (SAIL_38_G07) mutant plants were previously described (Suzuki et al. 2007). The *val1-5*, *val2-3* and *val1-5 val2-3*, as well as the *pVAL1:VAL1-GUS* were previously reported (Fouracre et al. 2021), as were also the *flc-3* allele, and the *bop1-5* (SAIL _14_C02), *bop2-2* (SALK-075879), and *bop1 bop2* mutant lines (Norberg et al. 2005). Genetic crosses were conducted to obtain the double and triple mutant lines described in this work. All the mutant lines were genotyped by PCR using the gene-specific primers listed in Supplementary Table S4.

Seeds were surface sterilized for 10 min using 75% (v/v) ethanol containing 0.01% Triton X-100, and then washed 3 times with ddH_2_O. Seeds were sown in plates containing 0.5× MS media with agar (without sucrose, with added vitamins), incubated 48 h at 4 °C for stratification, and then moved to 22 °C at either long-day (LD, 16 h in the light/8 h in the dark) or short-day conditions (SD, 8 h in the light/16 h in the dark). Seven-day-old seedlings were transferred from plates to soil.

### Flowering time analysis

Flowering time was scored by counting rosette leaf number at bolting (bolt = 2 cm) after moving imbibed seeds to the light at 22 °C. At least 10 plants were scored for each genotype and 2 or more biological repeats were performed for each experiment.

### GA_3_ treatment

Exogenous GA_3_ was applied as described previously (Yang et al. 2020) with some modifications. A stock solution of GA_3_ (Sigma) was prepared in 100% ethanol. For the treatment, 10 *μ*L of 100 *μ*M GA_3_ in 0.02% Silwet-77 was supplemented to the SAM of 1-wk-old seedlings twice per week. The volume of GA_3_ solution was progressively increased from 10 to 300 *μ*L with plant growth. Mock plants were treated with 1% (v/v) ethanol plus 0.02% Silwet-77. Exogenous GA_3_ treatment was applied until all treated plants had flowered. At least 10 plants were scored for each genotype and 2 biological repeats were performed for each experiment.

### GA_4_ content analysis

Dissected shoot apices (0.5 g) of 6-wk-old plants were collected, flash-frozen and ground into powder in liquid nitrogen. Samples were submitted to the Plant Hormone Quantification Service at IBMCP (Valencia, Spain) for GA_4_ quantification. Three biological replicates were analyzed for each genotype.

### Generation of Arabidopsis transgenic lines

Arabidopsis transgenic plants were generated by floral dip with *Agrobacterium tumefaciens*. The *pVAL1:VAL1-3xHA* binary vector generated in a previous work (Qüesta et al. 2016) was transformed into the *val1-2* mutant background for genetic complementation.

### GUS staining analysis

One-d-old germinated seeds, 7-d-old seedlings grown in LD, as well as 3- and 6-wk-old seedlings grown in SD were harvested for GUS staining according to (Li 2011). The protocol was modified according to the age of the plants. For young seedlings and 3-wk-old plants, 2 to 4 plants were collected in 2 mL tubes containing 1 mL staining buffer with fresh X-GUS. Samples were incubated overnight at 37 °C in the dark. Samples were subsequently washed with 2 mL of 75% ethanol for 30 min, and with 100% ethanol for 30 min. For the 6-wk-old plants, 1 seedling was stained in a 5 mL tube with 2 mL fresh staining buffer.

### Quantitative RT-qPCR analysis

Total RNA was extracted from dissected shoot apices or from whole seedlings using TRIzol Reagent (Thermo Fisher Scientific). Two microgram of total RNA was treated with DNase I at 37 °C for 1 h and reverse transcription was performed using Maxima RT (Thermo Fisher Scientific) with added the RiboLock RNase Inhibitor following the manufacturer instructions. RT-qPCR was performed in a Lightcycler 480 using the SYBR Green I Master Mix (Roche). *ACTIN2* (At3g18780), *UBIQUITIN-CONJUGATING ENZYME 21* (*UBC*, At5g25760), and *UBQ10* (At4g05320) were used as normalization gene control in all RT-qPCR experiments. At least 2 biological replicates were performed for each experiment. Six to nine technical repeats were performed for each sample. All RT-qPCR primer sequences are listed in Supplementary Table S4.

### RNA-Seq analysis

Total RNA was isolated from dissected shoot apices (0.5 g) from 6-wk-old plants grown under SD, using a RNeasy Plant Mini Kit (QIAGEN) and purified using an RNase-free DNase kit (QIAGEN). Extracted RNA was outsourced for library construction and sequencing on the Illumina platform with a 150 bp paired-end strategy.

The raw sequencing data was trimmed using *cutadapt* (Martin 2011) and mapped to the TAIR10 (Lamesch et al. 2012) genome version with *STAR* (Dobin et al. 2013) (see Supplementary Table S5 for read count statistics). Normalized bedGraph files were created with *STAR*, and gene counts computed with *stringtie* (Pertea et al. 2015) and the Araport11 (Cheng et al. 2017) gene annotations. The gene counts were collected using the *prepDE.py3* script from *stringtie*, and differential expression analysis performed using the *DEseq2* R package (Love et al. 2014). Volcano plots were generated using the *EnhancedVolcano* R package and principal component analysis (PCA) plots with base R functions. Gene ontology enrichment was performed using the Plant Regulomics webserver (Ran et al. 2020).

### ChIP analysis

Vegetative tissues (plant aerial part without roots) of 2-wk-old seedlings were harvested for ChIP experiment. At least 2 independent biological repeats were performed. ChIP experiments were performed as previously described (Yamaguchi et al. 2014) with some modifications to the protocol. 0.5 to 1 g of seedlings were harvested into a 50 mL tube containing 10 mL of 1% formaldehyde solution in 1× PBS buffer. Fixing solution was vacuum infiltrated during 15 min, following with the addition of 0.125 m glycine solution. Seedlings were washed twice with cold water then flash-frozen in liquid nitrogen. Frozen samples were ground to fine powder and resuspended in 5 mL of nuclei extraction buffer (100 mm MOPS pH 7.6, 10 mm MgCl_2_, 0.25 m sucrose, 5% Dextran T-40, 2.5% Ficoll 40) with freshly added 40 mm 2-mercaptoethanol and 1× protease inhibitor. Sample homogenate was filtered through 2 layers of Miracloth and then spun for 5 min at 10,000 × *g* at 4 °C. The nuclei enriched pellet was resuspended in 75 *μ*L of nuclei lysis buffer (50 mm Tris–HCl pH 8.0, 10 mm EDTA pH 8.0, 1% SDS). ChIP dilution buffer (625 *μ*L) “without Triton X-100” was added (16.7 mm Tris–HCl pH 8.0, 167 mm NaCl, 1.2 mm EDTA, 0.01% SDS) and the nuclei solution was transferred to a fresh 1.5 mL tube for chromatin sharing in a Bioruptor (Diagenode): 24 cycles, 30 s ON/30 s OFF in the low intensity setting. Samples were brought up to final volume of 900 *μ*L with ChIP dilution buffer “with Triton X-100” (16.7 mm Tris–HCl pH 8.0, 167 mm NaCl, 1.2 mm EDTA, 1.1% Triton X, 0.01% SDS), and 35 *μ*l 22% Triton X-100 were added. After spinning for 5 min at full speed, 15 *μ*L of magnetic protein A beads were added as preclearing with rotation at 4 °C for 2 h incubation. An aliquot of clear nuclei solution was stored (2% input). The remaining cleared nuclei solution was incubated with a suitable amount of antibody (4 *μ*L of H3K27me3 from Merck, 07-449, 5 *μ*L anti-HA from Abcam ab9110) rotating overnight at 4 °C. The protein/DNA complexes were captured by 15 *μ*L protein A magnetic beads for 4 h at 4 °C. Beads/protein/DNA complexes were washed twice with cold low salt wash buffer, high salt wash buffer, LiCl wash buffer, and 0.5× TE separately, and then lysed twice with 50 *μ*L nuclei lysis buffer at 65 °C for 30 min. Reverse crosslinking was performed by adding 6 *μ*L 5 m NaCl for 15 min at 95 °C with agitation (950 rpm) for both input and immunoprecipitation (IP) samples. DNA was purified by mixing the protein/DNA complex with an equal volume of phenol/chloroform. After spinning down during 5 min at maximum speed, the aqueous phase was transferred to a fresh tube. An equal volume of isopropanol with 1/10 of 5 m NaCl was added to each tube. Samples were incubated at room temperature for 30 min to precipitate DNA. Purified DNA was used for ChIP-qPCR analysis. *ACTIN2* and *SHOOT MERISTEMLESS* (*STM*, At1g62360) were used as controls in qPCR (Supplementary Table S4).

### Electrophoretic mobility shift assay

Expression and purification of GST-VAL1B3 and GST recombinant proteins were performed as previously described (Qüesta et al. 2016). EMSA was carried out using double-stranded fluorescently labeled DNA probes (TYE665-dsDNA). Double-stranded DNA probes were generated by annealing sense and antisense oligonucleotides (see the Supplementary Table S4 for sequences of oligonucleotides used). Annealing reactions were incubated for 3 min at 95 °C, 3 min at 72 °C, 3 min at 42 °C, and 5 min at 5 °C in a PCR thermocycler. Binding reaction conditions were as follows: 1× Binding buffer (10 mm Tris, 50 mm KCl, 1 mm DTT, pH7.5), 2.5% glycerol, 5 mm MgCl_2%_ and 0.025% NP-40 in a final volume of 20 *μ*L. Two microliter of 1 *μ*M TYE665-dsDNA and an equivalent volume of recombinant protein were used for the binding reaction. For the competition experiments, 25×–50× molar excess of unlabeled dsDNA probes were used. Binding reactions were incubated for 25 min at room temperature. Protein–DNA complexes were resolved on 1.6% Agarose gel in 0.5× TBE at 110 V for 30 min. TYE665-labeled DNA was detected using the Cy5-635 nm setting in the IQ800 system.

### In situ hybridization

RNA in situ hybridization probe was derived from a PCR product using primers oVB402 (GGAAATAGTTCAATCCCTCTG) and oVB403 (TAATACGACTCACTATAGG CTTGAGAAGGTAAAGGAGATG) using a T7 promoter in the reverse primer. *FD* RNA was labeled with digoxigenin. In situ hybridizations were performed as described (Balanzà et al. 2018). Fifteen-day-old plant apices were fixed for 2 h in FAE solution, dehydrated, embedded, and sectioned to 8 *µ*m. After dewaxing in histoclear and rehydrating, sections were treated for 20 min in 0.2 m HCl, neutralized for 10 min in 2× SSC and then incubated for 30 min with 1 *µ*g/mL Proteinase K at 37 °C. Proteinase action was blocked by treating with 2 mg/mL Gly for 5 min and post-fixation in 4% formaldehyde for 10 min. Subsequently, sections were dehydrated through an ethanol series before applying the hybridization solution (100 *µ*g/mL tRNA; 6× SSC; 3% SDS; 50% formamide, containing ∼100 ng/*μ*L of antisense DIG-labeled RNA probe), and left overnight at 52 °C. Then, sections were washed twice for 90 min in 2× SSC: formamide (50:50) at 52 °C before performing the antibody incubation and color detection.

### Statistical analysis

Statistical significance was determined by performing a One-way and two-way ANOVA analysis with multiple comparisons (*P*  *<*  *0.05*) using GraphPad software. Data were expressed as mean values ± SEM.

### Accession numbers

*VAL1* (AT2G30470), *VAL2* (AT4G32010), *FLC* (AT5G10140), *BOP1* (AT3G57130), *BOP2* (AT2G41370), *ATH1* (AT4G32980), *FT* (AT1G65480), *FD* (AT4G35900), *SOC1* (AT2G45660), *FUL* (AT5G60910), *LFY* (AT5G61850), *GAI* (AT1G14920), *GA3OX1* (AT1G15550), *GA20OX1* (AT4G25420), *GA20OX2* (AT5G51810), *GA2OX2* (AT1G30040), *GA2OX6* (AT1G02400).

Sequence data from this article can be found in the Gene Expression Omnibus (GEO) database under accession number GSE272866.

## Supplementary Material

kiaf160_Supplementary_Data

## Data Availability

The data underlying this article are available in the Gene Expression Omnibus (GEO) database and can be accessed with the accession number GSE272866. This manuscript does not report any original code.

## Supplementary data

The following materials are available in the online version of this article.

**Supplementary Figure S1.** Characterization of *VAL1* complementation lines.

**Supplementary Figure S2.** VAL1 regulates flowering time besides of *FLC* pathway.

**Supplementary Figure S3.** Exogenous GA_3_ rescues the late flowering phenotype of *val1* mutant under SD.

**Supplementary Figure S4.** Transcriptome changes in *val1* and *val2* mutant.

**Supplementary Figure S5.** VAL1 regulates the transcription level of flowering and GA pathway genes.

**Supplementary Figure S6.** VAL1 induces repression of *BOP1* and *BOP2* via the PRC2 pathway.

**Supplementary Table S1.** Nonredundant GO-terms for genes up- and downregulated in *val1* mutant.

**Supplementary Table S2.** Flowering related terms identified in the list of *val1*_only_UP genes.

**Supplementary Table S3.** Data included in heat-map of Fig. 4E.

**Supplementary Table S4.** List of primers used in this study.

**Supplementary Table S5.** RNA-seq statistics.

**Supplementary Data Set 1.** RNA-seq differential expression analysis.

**Supplementary Data Set 2.** Genes uniquely up- or downregulated in either *val1* or *val2* mutant (non-overlaps).

**Supplementary Data Set 3.** Gene ontology (GO) analysis in genes upregulated in *val1* mutant (VAL1_only_UP).

**Supplementary Data Set 4.** Gene ontology (GO) analysis in genes downregulated in *val1* mutant (VAL1_only_DOWN).

## Dive Curated Terms

The following phenotypic, genotypic, and functional terms are of significance to the work described in this paper:

MAF4 Gramene: AT5G65070
MAF4 Araport: AT5G65070

MAF5 Gramene: AT5G65080
MAF5 Araport: AT5G65080

AGL15 Gramene: AT5G13790
AGL15 Araport: AT5G13790

AGL24 Gramene: AT4G24540
AGL24 Araport: AT4G24540

MIR157C Gramene: AT3G18217
MIR157C Araport: AT3G18217

SPL9 Gramene: At2g42200
SPL9 Araport: At2g42200

BOP1 Gramene: AT3G57130
BOP1 Araport: AT3G57130

GLK2 Gramene: AT5G44190
GLK2 Araport: AT5G44190

GA20ox1 Gramene: At4g25420
GA20ox1 Araport: At4g25420

GA20ox2 Gramene: AT5G51810
GA20ox2 Araport: AT5G51810

GA2ox6 Gramene: AT1G02400
GA2ox6 Araport: AT1G02400

GA3ox1 Gramene: At1g15550
GA3ox1 Araport: At1g15550

VAL1 Gramene: AT2G30470
VAL1 Araport: AT2G30470

SPL13A Gramene: At5g50570
SPL13A Araport: At5g50570

SPL13B Gramene: At5g50670
SPL13B Araport: At5g50670

FLC Gramene: AT5G10140
FLC Araport: AT5G10140

BOP2 Gramene: AT2G41370
BOP2 Araport: AT2G41370

FT Gramene: AT1G65480
FT Araport: AT1G65480

FUL Gramene: AT5G60910
FUL Araport: AT5G60910

FD Gramene: AT4G35900
FD Araport: AT4G35900

LFY Gramene: AT5G61850
LFY Araport: AT5G61850

VAL2 Gramene: AT4G32010
VAL2 Araport: AT4G32010

ATH1 Gramene: AT4G32980
ATH1 Araport: AT4G32980

SOC1 Gramene: AT2G45660
SOC1 Araport: AT2G45660

GAI Gramene: AT1G14920
GAI Araport: AT1G14920

## References

[kiaf160-B1] Abe M, Kobayashi Y, Yamamoto S, Daimon Y, Yamaguchi A, Ikeda Y, Ichinoki H, Notaguchi M, Goto K, Araki T. FD, a bZIP protein mediating signals from the floral pathway integrator FT at the shoot apex. Science. 2005:309(5737):1052–1056. 10.1126/science.1115983 16099979

[kiaf160-B2] Andrés F, Coupland G. The genetic basis of flowering responses to seasonal cues. Nat Rev Genet. 2012:13(9):627–639. 10.1038/nrg3291 22898651

[kiaf160-B3] Andrés F, Romera-Branchat M, Martínez-Gallegos R, Patel V, Schneeberger K, Jang S, Altmüller J, Nürnberg P, Coupland G. Floral induction in Arabidopsis by FLOWERING LOCUS T requires direct repression of BLADE-ON-PETIOLE genes by the homeodomain protein PENNYWISE. Plant Physiol. 2015:169(3):2187–2199. 10.1104/pp.15.00960 26417007 PMC4634070

[kiaf160-B4] Axtell MJ, Bartel DP. Antiquity of MicroRNAs and their targets in land plants. Plant Cell. 2005:17(6):1658–1673. 10.1105/tpc.105.032185 15849273 PMC1143068

[kiaf160-B5] Balanzà V, Martínez-Fernández I, Sato S, Yanofsky MF, Kaufmann K, Angenent GC, Bemer M, Ferrándiz C. Genetic control of meristem arrest and life span in Arabidopsis by a FRUITFULL-APETALA2 pathway. Nat Commun. 2018:9(1):565. 10.1038/s41467-018-03067-5 29422669 PMC5805735

[kiaf160-B6] Berry S, Dean C. Environmental perception and epigenetic memory: mechanistic insight through FLC. Plant J. 2015:83(1):133–148. 10.1111/tpj.12869 25929799 PMC4691321

[kiaf160-B7] Capovilla G, Pajoro A, Immink RG, Schmid M. Role of alternative pre-mRNA splicing in temperature signaling. Curr Opin Plant Biol. 2015:27:97–103. 10.1016/j.pbi.2015.06.016 26190743

[kiaf160-B8] Chan KX, Phua SY, Crisp P, McQuinn R, Pogson BJ. Learning the languages of the chloroplast: retrograde signaling and beyond. Annu Rev Plant Biol. 2016:67(1):25–53. 10.1146/annurev-arplant-043015-111854 26735063

[kiaf160-B9] Chen N, Veerappan V, Abdelmageed H, Kang M, Allen RD. HSI2/VAL1 silences AGL15 to regulate the developmental transition from seed maturation to vegetative growth in arabidopsis. Plant Cell. 2018:30(3):600–619. 10.1105/tpc.17.00655 29475938 PMC5894832

[kiaf160-B10] Cheng C-Y, Krishnakumar V, Chan AP, Thibaud-Nissen F, Schobel S, Town CD. Araport11: a complete reannotation of the Arabidopsis thaliana reference genome. Plant J. 2017:89(4):789–804. 10.1111/tpj.13415 27862469

[kiaf160-B11] Collani S, Neumann M, Yant L, Schmid M. FT modulates genome-wide DNA-binding of the bZIP transcription factor FD. Plant Physiol. 2019:180(1):367–380. 10.1104/pp.18.01505 30770462 PMC6501114

[kiaf160-B12] Conti L . Hormonal control of the floral transition: can one catch them all? Dev Biol. 2017:430(2):288–301. 10.1016/j.ydbio.2017.03.024 28351648

[kiaf160-B13] Couzigou J-M, Mondy S, Sahl L, Gourion B, Ratet P. To be or not to be: evolutionary tinkering for symbiotic organ identity. Plant Signal Behav. 2013:8(8):e24969. 10.4161/psb.24969 23733067 PMC4004616

[kiaf160-B14] Crick J, Corrigan L, Belcram K, Khan M, Dawson JW, Adroher B, Li S, Hepworth SR, Pautot V. Floral organ abscission in Arabidopsis requires the combined activities of three TALE homeodomain transcription factors. J Exp Bot. 2022:73(18):6150–6169. 10.1093/jxb/erac255 35689803

[kiaf160-B15] Davière J-M, Achard P. Gibberellin signaling in plants. Development. 2013:140(6):1147–1151. 10.1242/dev.087650 23444347

[kiaf160-B16] Dobin A, Davis CA, Schlesinger F, Drenkow J, Zaleski C, Jha S, Batut P, Chaisson M, Gingeras TR. STAR: ultrafast universal RNA-seq aligner. Bioinformatics. 2013:29(1):15–21. 10.1093/bioinformatics/bts635 23104886 PMC3530905

[kiaf160-B17] Ejaz M, Bencivenga S, Tavares R, Bush M, Sablowski R. ARABIDOPSIS THALIANA HOMEOBOX GENE 1 controls plant architecture by locally restricting environmental responses. Proc Natl Acad Sci U S A. 2021:118(17):e2018615118. 10.1073/pnas.2018615118 33888582 PMC8092594

[kiaf160-B18] Fouracre JP, He J, Chen VJ, Sidoli S, Poethig RS. VAL genes regulate vegetative phase change via miR156-dependent and independent mechanisms. PLoS Genet. 2021:17(6):e1009626. 10.1371/journal.pgen.1009626 34181637 PMC8270478

[kiaf160-B19] Galvão VC, Horrer D, Küttner F, Schmid M. Spatial control of flowering by DELLA proteins in Arabidopsis thaliana. Development. 2012:139(21):4072–4082. 10.1242/dev.080879 22992955

[kiaf160-B20] Ha CM, Jun JH, Nam HG, Fletcher JC. BLADE-ON-PETIOLE1 encodes a BTB/POZ domain protein required for leaf morphogenesis in Arabidopsis thaliana. Plant Cell Physiol. 2004:45(10):1361–1370. 10.1093/pcp/pch201 15564519

[kiaf160-B21] Ha CM, Jun JH, Nam HG, Fletcher JC. BLADE-ON-PETIOLE1 and 2 control Arabidopsis lateral organ fate through regulation of LOB domain and adaxial-abaxial polarity genes. Plant Cell. 2007:19(6):1809–1825. 10.1105/tpc.107.051938 17601823 PMC1955725

[kiaf160-B22] Harberd NP . Relieving DELLA restraint. Science. 2003:299(5614):1853–1854. 10.1126/science.1083217 12649470

[kiaf160-B23] He J, Xu M, Willmann MR, McCormick K, Hu T, Yang L, Starker CG, Voytas DF, Meyers BC, Poethig RS. Threshold-dependent repression of SPL gene expression by miR156/miR157 controls vegetative phase change in Arabidopsis thaliana. PLoS Genet. 2018:14(4):e1007337. 10.1371/journal.pgen.1007337 29672610 PMC5929574

[kiaf160-B24] He Y, Chen T, Zeng X. Genetic and epigenetic understanding of the seasonal timing of flowering. Plant Commun. 2020:1(1):100008. 10.1016/j.xplc.2019.100008 33404547 PMC7747966

[kiaf160-B25] Hepworth J, Dean C. Flowering locus C's lessons: conserved chromatin switches underpinning developmental timing and adaptation. Plant Physiol. 2015:168(4):1237–1245. 10.1104/pp.15.00496 26149571 PMC4528751

[kiaf160-B26] Hepworth SR, Zhang Y, McKim S, Li X, Haughn GW. BLADE-ON-PETIOLE–dependent signaling controls leaf and floral patterning in Arabidopsis. Plant Cell. 2005:17(5):1434–1448. 10.1105/tpc.104.030536 15805484 PMC1091766

[kiaf160-B27] Hernández-Verdeja T, Strand Å. Retrograde signals navigate the path to chloroplast development. Plant Physiol. 2018:176(2):967–976. 10.1104/pp.17.01299 29254985 PMC5813530

[kiaf160-B28] Hu T, Manuela D, Xu M. SQUAMOSA PROMOTER BINDING PROTEIN-LIKE 9 and 13 repress BLADE-ON-PETIOLE 1 and 2 directly to promote adult leaf morphology in Arabidopsis. J Exp Bot. 2023:74(6):1926–1939. 10.1093/jxb/erad017 36629519 PMC10049914

[kiaf160-B29] Izawa T . What is going on with the hormonal control of flowering in plants? Plant J. 2021:105(2):431–445. 10.1111/tpj.15036 33111430

[kiaf160-B30] Jaeger KE, Pullen N, Lamzin S, Morris RJ, Wigge PA. Interlocking feedback loops govern the dynamic behavior of the floral transition in Arabidopsis. Plant Cell. 2013:25(3):820–833. 10.1105/tpc.113.109355 23543784 PMC3634691

[kiaf160-B31] Jin S, Ahn JH. Regulation of flowering time by ambient temperature: repressing the repressors and activating the activators. New Phytol. 2021:230(3):938–942. 10.1111/nph.17217 33474759

[kiaf160-B32] Jing Y, Guo Q, Lin R. The B3-domain transcription factor VAL1 regulates the floral transition by repressing *FLOWERING LOCUS T*. Plant Physiol. 2019:181:236–248. 10.1104/pp.19.00642 31289216 PMC6716252

[kiaf160-B33] Karim MR, Hirota A, Kwiatkowska D, Tasaka M, Aida M. A role for Arabidopsis PUCHI in floral meristem identity and bract suppression. Plant Cell. 2009:21(5):1360–1372. 10.1105/tpc.109.067025 19482972 PMC2700531

[kiaf160-B34] Khan M, Ragni L, Tabb P, Salasini BC, Chatfield S, Datla R, Lock J, Kuai X, Després C, Proveniers M, et al Repression of lateral organ boundary genes by PENNYWISE and POUND-FOOLISH is essential for meristem maintenance and flowering in Arabidopsis. Plant Physiol. 2015:169(3):2166–2186. 10.1104/pp.15.00915 26417006 PMC4634066

[kiaf160-B35] Khan M, Tabb P, Hepworth SR. BLADE-ON-PETIOLE1 and 2 regulate Arabidopsis inflorescence architecture in conjunction with homeobox genes KNAT6 and ATH1. Plant Signal Behav. 2012:7(7):788–792. 10.4161/psb.20599 22751300 PMC3583964

[kiaf160-B36] Kinoshita A, Richter R. Genetic and molecular basis of floral induction in Arabidopsis thaliana. J Exp Bot. 2020:71(9):2490–2504. 10.1093/jxb/eraa057 32067033 PMC7210760

[kiaf160-B37] Lamesch P, Berardini TZ, Li D, Swarbreck D, Wilks C, Sasidharan R, Muller R, Dreher K, Alexander DL, Garcia-Hernandez M, et al The Arabidopsis information resource (TAIR): improved gene annotation and new tools. Nucleic Acids Res. 2012:40(D1):D1202–D1210. 10.1093/nar/gkr1090 22140109 PMC3245047

[kiaf160-B38] Langridge J . Effect of day-length and gibberellic acid on the flowering of Arabidopsis. Nature. 1957:180(4575):36–37. 10.1038/180036a0

[kiaf160-B39] Li X . Histostaining for tissue expression pattern of promoter-driven GUS activity in Arabidopsis. Bio-protocols. 2011. 10.21769/BioProtoc.93

[kiaf160-B40] Love MI, Huber W, Anders S. Moderated estimation of fold change and dispersion for RNA-seq data with DESeq2. Genome Biol. 2014:15(12):550. 10.1186/s13059-014-0550-8 25516281 PMC4302049

[kiaf160-B41] Martin M . Cutadapt removes adapter sequences from high-throughput sequencing reads. EMBnet J. 2011:17(1):10–12. 10.14806/ej.17.1.200

[kiaf160-B42] Mateos JL, Madrigal P, Tsuda K, Rawat V, Richter R, Romera-Branchat M, Fornara F, Schneeberger K, Krajewski P, Coupland G. Combinatorial activities of SHORT VEGETATIVE PHASE and FLOWERING LOCUS C define distinct modes of flowering regulation in Arabidopsis. Genome Biol. 2015:16(1):31. 10.1186/s13059-015-0597-1 25853185 PMC4378019

[kiaf160-B43] McKim SM, Stenvik G-E, Butenko MA, Kristiansen W, Cho SK, Hepworth SR, Aalen RB, Haughn GW. The BLADE-ON-PETIOLE genes are essential for abscission zone formation in Arabidopsis. Development. 2008:135(8):1537–1546. 10.1242/dev.012807 18339677

[kiaf160-B44] Mikulski P, Wolff P, Lu T, Nielsen M, Echevarria EF, Zhu D, Questa JI, Saalbach G, Martins C, Dean C. VAL1 acts as an assembly platform co-ordinating co-transcriptional repression and chromatin regulation at Arabidopsis FLC. Nat Commun. 2022:13(1):5542. 10.1038/s41467-022-32897-7 36130923 PMC9492735

[kiaf160-B45] Murase K, Hirano Y, Sun T, Hakoshima T. Gibberellin-induced DELLA recognition by the gibberellin receptor GID1. Nature. 2008:456(7221):459–463. 10.1038/nature07519 19037309

[kiaf160-B46] Norberg M, Holmlund M, Nilsson O. The BLADE ON PETIOLE genes act redundantly to control the growth and development of lateral organs. Development. 2005:132(9):2203–2213. 10.1242/dev.01815 15800002

[kiaf160-B47] Pertea M, Pertea GM, Antonescu CM, Chang T-C, Mendell JT, Salzberg SL. StringTie enables improved reconstruction of a transcriptome from RNA-seq reads. Nat Biotechnol. 2015:33(3):290–295. 10.1038/nbt.3122 25690850 PMC4643835

[kiaf160-B48] Picó S, Ortiz-Marchena MI, Merini W, Calonje M. Deciphering the role of POLYCOMB REPRESSIVE COMPLEX1 variants in regulating the acquisition of flowering competence in Arabidopsis. Plant Physiol. 2015:168(4):1286–1297. 10.1104/pp.15.00073 25897002 PMC4528732

[kiaf160-B49] Porri A, Torti S, Romera-Branchat M, Coupland G. Spatially distinct regulatory roles for gibberellins in the promotion of flowering of Arabidopsis under long photoperiods. Development. 2012:139(12):2198–2209. 10.1242/dev.077164 22573618

[kiaf160-B50] Proveniers M, Rutjens B, Brand M, Smeekens S. The Arabidopsis TALE homeobox gene ATH1 controls floral competency through positive regulation of FLC. Plant J. 2007:52(5):899–913. 10.1111/j.1365-313X.2007.03285.x 17908157

[kiaf160-B51] Qüesta JI, Song J, Geraldo N, An H, Dean C. Arabidopsis transcriptional repressor VAL1 triggers Polycomb silencing at FLC during vernalization. Science. 2016:353(6298):485–488. 10.1126/science.aaf7354 27471304

[kiaf160-B52] Ran X, Zhao F, Wang Y, Liu J, Zhuang Y, Ye L, Qi M, Cheng J, Zhang Y. Plant regulomics: a data-driven interface for retrieving upstream regulators from plant multi-omics data. Plant J. 2020:101(1):237–248. 10.1111/tpj.14526 31494994

[kiaf160-B53] Ratcliffe OJ, Kumimoto RW, Wong BJ, Riechmann JL. Analysis of the Arabidopsis MADS AFFECTING FLOWERING gene family: MAF2 prevents vernalization by short periods of cold [W]. Plant Cell. 2003:15(5):1159–1169. 10.1105/tpc.009506 12724541 PMC153723

[kiaf160-B54] Schwab R, Palatnik JF, Riester M, Schommer C, Schmid M, Weigel D. Specific effects of MicroRNAs on the plant transcriptome. Dev Cell. 2005:8(4):517–527. 10.1016/j.devcel.2005.01.018 15809034

[kiaf160-B55] Searle I, He Y, Turck F, Vincent C, Fornara F, Kröber S, Amasino RA, Coupland G. The transcription factor FLC confers a flowering response to vernalization by repressing meristem competence and systemic signaling in Arabidopsis. Genes Dev. 2006:20(7):898–912. 10.1101/gad.373506 16600915 PMC1472290

[kiaf160-B56] Shen L, Thong Z, Gong X, Shen Q, Gan Y, Yu H. The putative PRC1 RING-finger protein AtRING1A regulates flowering through repressing MADS AFFECTING FLOWERING genes in Arabidopsis. Development. 2014:141(6):1303–1312. 10.1242/dev.104513 24553292

[kiaf160-B57] Silverstone AL, Jung H-S, Dill A, Kawaide H, Kamiya Y, Sun T. Repressing a repressor: gibberellin-induced rapid reduction of the RGA protein in Arabidopsis. Plant Cell. 2001:13(7):1555–1566. 10.1105/tpc.010047 11449051 PMC139546

[kiaf160-B58] Song YH, Shim JS, Kinmonth-Schultz HA, Imaizumi T. Photoperiodic flowering: time measurement mechanisms in leaves. Annu Rev Plant Biol. 2015:66(1):441–464. 10.1146/annurev-arplant-043014-115555 25534513 PMC4414745

[kiaf160-B59] Susila H, Nasim Z, Gawarecka K, Jung J-Y, Jin S, Youn G, Ahn JH. Chloroplasts prevent precocious flowering through a *GOLDEN2-LIKE–B-BOX DOMAIN PROTEIN* module. Plant Commun. 2023:4(3):100515. 10.1016/j.xplc.2023.100515 36597356 PMC10203396

[kiaf160-B60] Suzuki M, Wang HH, McCarty DR. Repression of the LEAFY COTYLEDON 1/B3 regulatory network in plant embryo development by VP1/ABSCISIC ACID INSENSITIVE 3-LIKE B3 genes. Plant Physiol. 2007:143(2):902–911. 10.1104/pp.106.092320 17158584 PMC1803726

[kiaf160-B61] Tsukagoshi H, Morikami A, Nakamura K. Two B3 domain transcriptional repressors prevent sugar-inducible expression of seed maturation genes in Arabidopsis seedlings. Proc Natl Acad Sci U S A. 2007:104(7):2543–2547. 10.1073/pnas.0607940104 17267611 PMC1785360

[kiaf160-B62] Wahl V, Ponnu J, Schlereth A, Arrivault S, Langenecker T, Franke A, Feil R, Lunn JE, Stitt M, Schmid M. Regulation of flowering by trehalose-6-phosphate signaling in Arabidopsis thaliana. Science. 2013:339(6120):704–707. 10.1126/science.1230406 23393265

[kiaf160-B63] Wang J-W, Czech B, Weigel D. miR156-regulated SPL transcription factors define an endogenous flowering pathway in Arabidopsis thaliana. Cell. 2009:138(4):738–749. 10.1016/j.cell.2009.06.014 19703399

[kiaf160-B64] Wang Y, Salasini BC, Khan M, Devi B, Bush M, Subramaniam R, Hepworth SR. Clade I TGACG-motif binding basic leucine zipper transcription factors mediate BLADE-ON-PETIOLE-dependent regulation of development. Plant Physiol. 2019:180(2):937–951. 10.1104/pp.18.00805 30923069 PMC6548253

[kiaf160-B65] Wigge PA, Kim MC, Jaeger KE, Busch W, Schmid M, Lohmann JU, Weigel D. Integration of spatial and temporal information during floral induction in Arabidopsis. Science. 2005:309(5737):1056–1059. 10.1126/science.1114358 16099980

[kiaf160-B66] Willige BC, Ghosh S, Nill C, Zourelidou M, Dohmann EMN, Maier A, Schwechheimer C. The DELLA domain of GA INSENSITIVE mediates the interaction with the GA INSENSITIVE DWARF1A gibberellin receptor of Arabidopsis. Plant Cell. 2007:19(4):1209–1220. 10.1105/tpc.107.051441 17416730 PMC1913748

[kiaf160-B67] Wilson RN, Heckman JW, Somerville CR. Gibberellin is required for flowering in Arabidopsis thaliana under short days 1. Plant Physiol. 1992:100(1):403–408. 10.1104/pp.100.1.403 16652976 PMC1075565

[kiaf160-B68] Wu G, Park MY, Conway SR, Wang J-W, Weigel D, Poethig RS. The sequential action of miR156 and miR172 regulates developmental timing in Arabidopsis. Cell. 2009:138(4):750–759. 10.1016/j.cell.2009.06.031 19703400 PMC2732587

[kiaf160-B69] Xu M, Hu T, McKim SM, Murmu J, Haughn GW, Hepworth SR. Arabidopsis BLADE-ON-PETIOLE1 and 2 promote floral meristem fate and determinacy in a previously undefined pathway targeting APETALA1 and AGAMOUS-LIKE24. Plant J. 2010:63(6):974–989. 10.1111/j.1365-313X.2010.04299.x 20626659

[kiaf160-B70] Xu M, Hu T, Smith MR, Poethig RS. Epigenetic regulation of vegetative phase change in Arabidopsis. Plant Cell. 2016a:28(1):28–41. 10.1105/tpc.15.00854 26704382 PMC4746683

[kiaf160-B71] Xu M, Hu T, Zhao J, Park M-Y, Earley KW, Wu G, Yang L, Poethig RS. Developmental functions of miR156-regulated SQUAMOSA PROMOTER BINDING PROTEIN-LIKE (SPL) genes in Arabidopsis thaliana. PLoS Genet. 2016b:12(8):e1006263. 10.1371/journal.pgen.1006263 27541584 PMC4991793

[kiaf160-B72] Yamaguchi A, Wu M-F, Yang L, Wu G, Poethig RS, Wagner D. The microRNA-regulated SBP-box transcription factor SPL3 is a direct upstream activator of LEAFY, FRUITFULL, and APETALA1. Dev Cell. 2009:17(2):268–278. 10.1016/j.devcel.2009.06.007 19686687 PMC2908246

[kiaf160-B73] Yamaguchi N, Winter CM, Wu M-F, Kwon CS, William DA, Wagner D. PROTOCOLS: chromatin immunoprecipitation from Arabidopsis tissues. Arabidopsis Book. 2014:12:e0170. 10.1199/tab.0170 24653666 PMC3952383

[kiaf160-B74] Yang C, Bratzel F, Hohmann N, Koch M, Turck F, Calonje M. VAL- and AtBMI1-mediated H2Aub initiate the switch from embryonic to postgerminative growth in Arabidopsis. Curr Biol. 2013a:23(14):1324–1329. 10.1016/j.cub.2013.05.050 23810531

[kiaf160-B75] Yang L, Xu M, Koo Y, He J, Poethig RS. Sugar promotes vegetative phase change in Arabidopsis thaliana by repressing the expression of MIR156A and MIR156C. eLife. 2013b:2:e00260. 10.7554/eLife.00260 23538384 PMC3608266

[kiaf160-B76] Yang T, Sun Y, Wang Y, Zhou L, Chen M, Bian Z, Lian Y, Xuan L, Yuan G, Wang X, et al AtHSPR is involved in GA- and light intensity-mediated control of flowering time and seed set in Arabidopsis. J Exp Bot. 2020:71(12):3543–3559. 10.1093/jxb/eraa128 32157303 PMC7475253

[kiaf160-B77] Yu S, Cao L, Zhou CM, Zhang TQ, Lian H, Sun Y, Wu J, Huang J, Wang G, Wang JW. Sugar is an endogenous cue for juvenile-to-adult phase transition in plants. eLife. 2013:2:e00269. 10.7554/eLife.00269 23543845 PMC3610343

[kiaf160-B78] Yuan L, Song X, Zhang L, Yu Y, Liang Z, Lei Y, Ruan J, Tan B, Liu J, Li C. The transcriptional repressors VAL1 and VAL2 recruit PRC2 for genome-wide polycomb silencing in Arabidopsis. Nucleic Acids Res. 2021:49(1):98–113. 10.1093/nar/gkaa1129 33270882 PMC7797069

[kiaf160-B79] Yuan W, Luo X, Li Z, Yang W, Wang Y, Liu R, Du J, He Y. A cis cold memory element and a trans epigenome reader mediate Polycomb silencing of FLC by vernalization in Arabidopsis. Nat Genet. 2016:48(12):1527–1534. 10.1038/ng.3712 27819666

[kiaf160-B80] Zhao J, Doody E, Poethig RS. Reproductive competence is regulated independently of vegetative phase change in Arabidopsis thaliana. Curr Biol. 2023:33(3):487–497.e2. 10.1016/j.cub.2022.12.029 36634678 PMC9905307

