# Supplementary material for: The transcriptional regulator VAL1 promotes Arabidopsis flowering by repressing the organ boundary genes BOP1 and BOP2
Source: Plant Physiol. 2025 Apr 24;198(2):kiaf160. doi: 10.1093/plphys/kiaf160 (PMC12152480; doi:10.1093/plphys/kiaf160)
Supplement: kiaf160_Supplementary_Data [file kiaf160_supplementary_data.zip › Supplementary Figures 1-6 accepted_compressed.pdf]

Figure S1

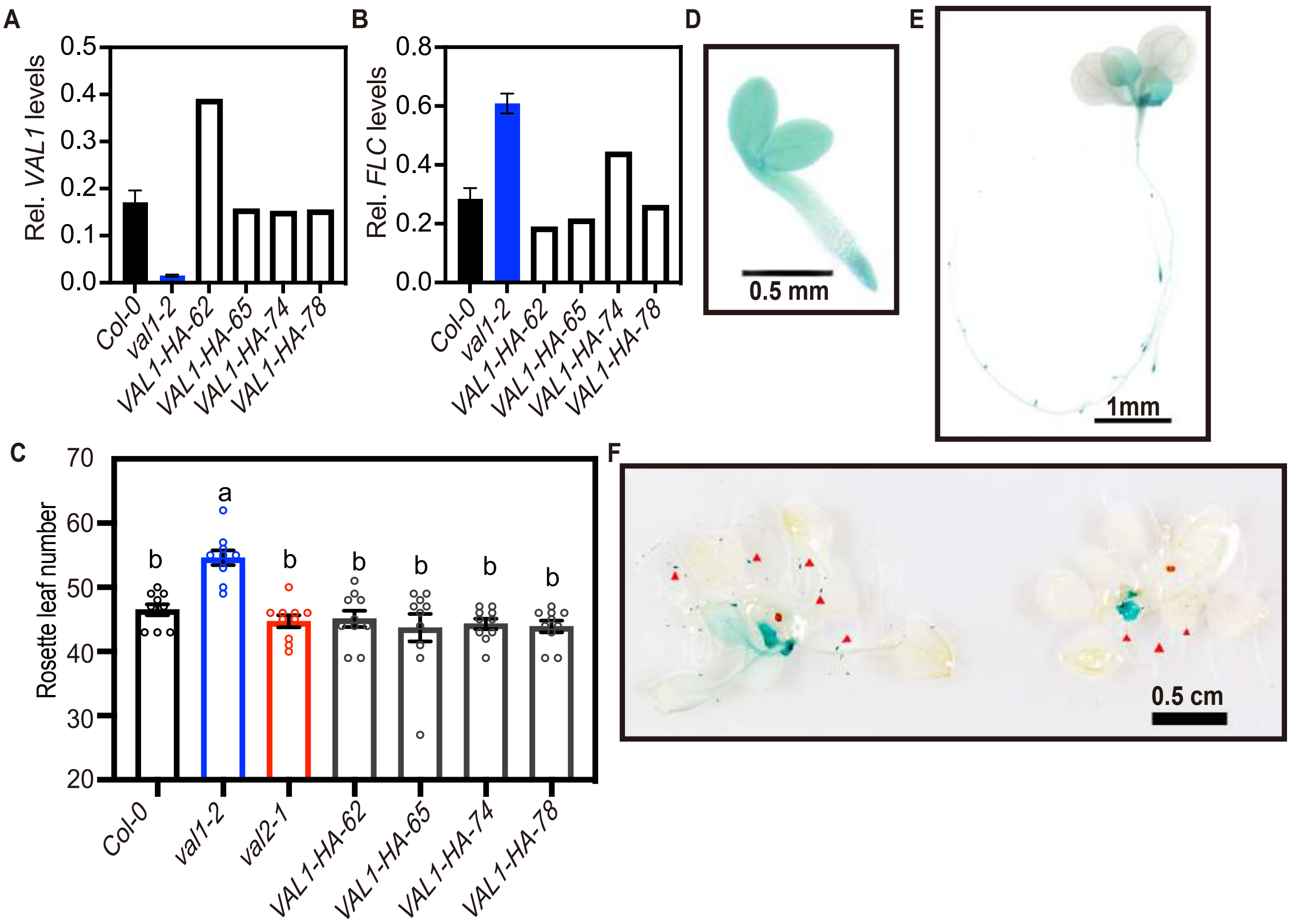

Supplementary Figure S1 Characterization of *VAL1* complementation lines.
(A, B) Relative (Rel.) transcript levels of *VAL1* (A) and *FLC* (B) mRNA levels in Col-0 (WT), *val1* and in four *pVAL1:VAL1-3xHA* complementation lines assessed by RT-qPCR. *UBC* was used as normalization control. Values are means ±SEM of three biological replicates for WT and *val1*. Only one biological replicate was used for each of the *pVAL1:VAL1-3xHA* transgenic lines.
(C) Rosette leaf number of WT, *val1*, *val2* and four *VAL1* complementation lines (*pVAL1:VAL1-3xHA*) grown under short days (SDs) (n=10). Different letters indicate significant differences as determined using Ordinary one-way ANOVA Multiple comparisons (p<0.05).
(D-F) Expression of pVAL1:VAL1-GUS in 1-day germinated seeds in long days (LDs) (D, scale bar is 0.5 mm), 1-week-old seedling in LDs (E, scale bar is 1 mm) and 3-week-old seedlings grown in SDs (F, scale bar is 0.5 cm). Red triangles in (F) indicate VAL1-GUS expression in the root tips, and two single plants show the same results.

Figure S2

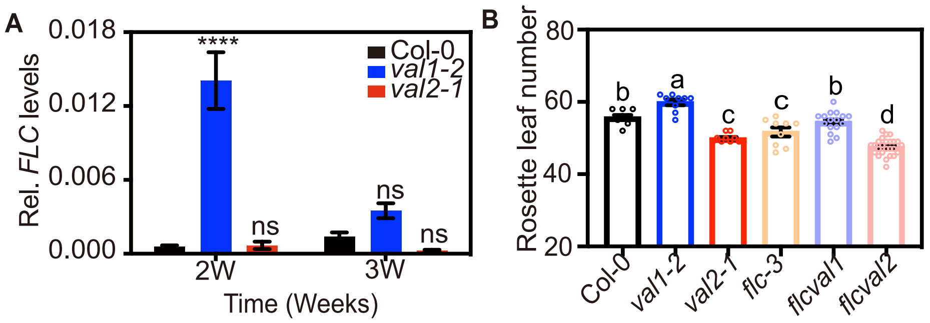

Supplementary Figure S2 VAL1 regulates flowering time besides of *FLC* pathway.

(A) Relative (Rel.) *FLC* transcript abundance tested by RT-qPCR in Col-0 (WT), *val1* and *val2* mutant plants grown under long days (LDs). Seeds were stratified during 72 h in the dark at 4°C, and then transferred to the light for germination at 22°C in LDs. Plants were incubated during 2 and 3 weeks (2W and 3W) before sample collection. Leaf material was collected for total RNA isolation. Values are means ±SEM (n=6). *ACTIN2* was used as normalization control. Different stars indicate significant differences as determined using Ordinary two-way ANOVA Multiple comparisons (****p<0.0001, ns, not significant).

(B) Flowering time of WT, *val1*, *val2* and *flc-3* single mutants, and *flcval1* and *flcval2* double mutant plants grown under short days (SDs), measured as rosette leaf number. Values are means ±SEM (n≥9). Different letters indicate significant differences as determined using Ordinary one-way ANOVA Multiple comparisons (p<0.05).

The experiment described in this figure is an independent replicate of the experiment presented in Figure 2B.

Figure S3

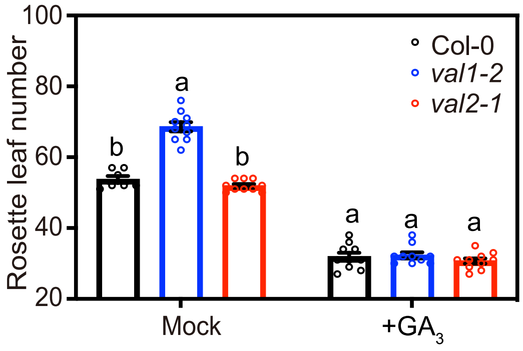

Supplementary Figure S3 Exogenous gibberellin 3 (GA_3) rescues the late flowering phenotype of *val1* mutant under SDs.

Rosette leaf number at flowering measured for Col-0 (WT), *val1* and *val2* mutant plants grown under short days (SDs) with or without 100 uM GA_3 treatment. Values are means ±SEM (n≥7). This is an independent replicate of the experiment described in Figure 3B. Different letters indicate significant differences as determined using Ordinary two-way ANOVA Multiple comparisons (p<0.05).

Figure S4

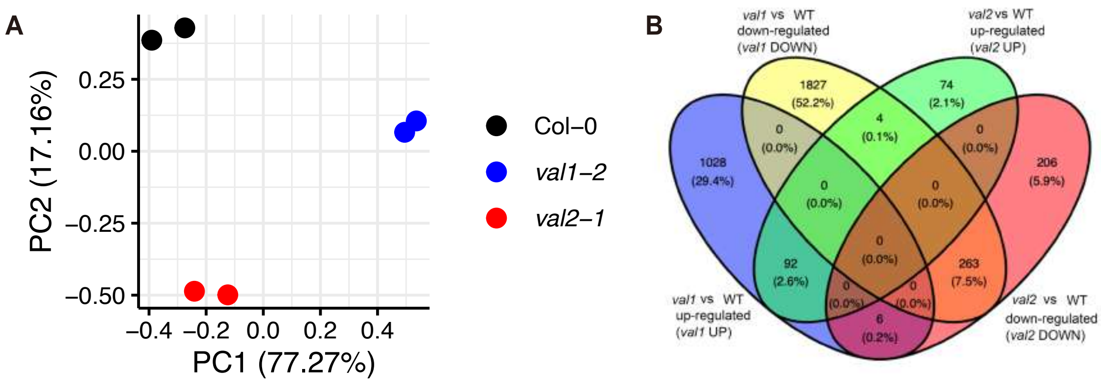

Supplementary Figure S4 Transcriptome changes in *val1* and *val2* mutant.
(A) Principal component analysis (PCA) of RNA-seq samples. RNA-seq was performed using apices isolated from *val1*, *val2* and Col-0 (WT) plants. 2 biological replicates were performed for each line.
(B) Venn diagram shows overlaps of differentially expressed genes (DEGs) in *val1* and *val2* vs. WT. The number of overlapped and non-overlapped DEGs are indicated. Percentages in parentheses were calculated with the total numbers of DEGs.

Figure S5

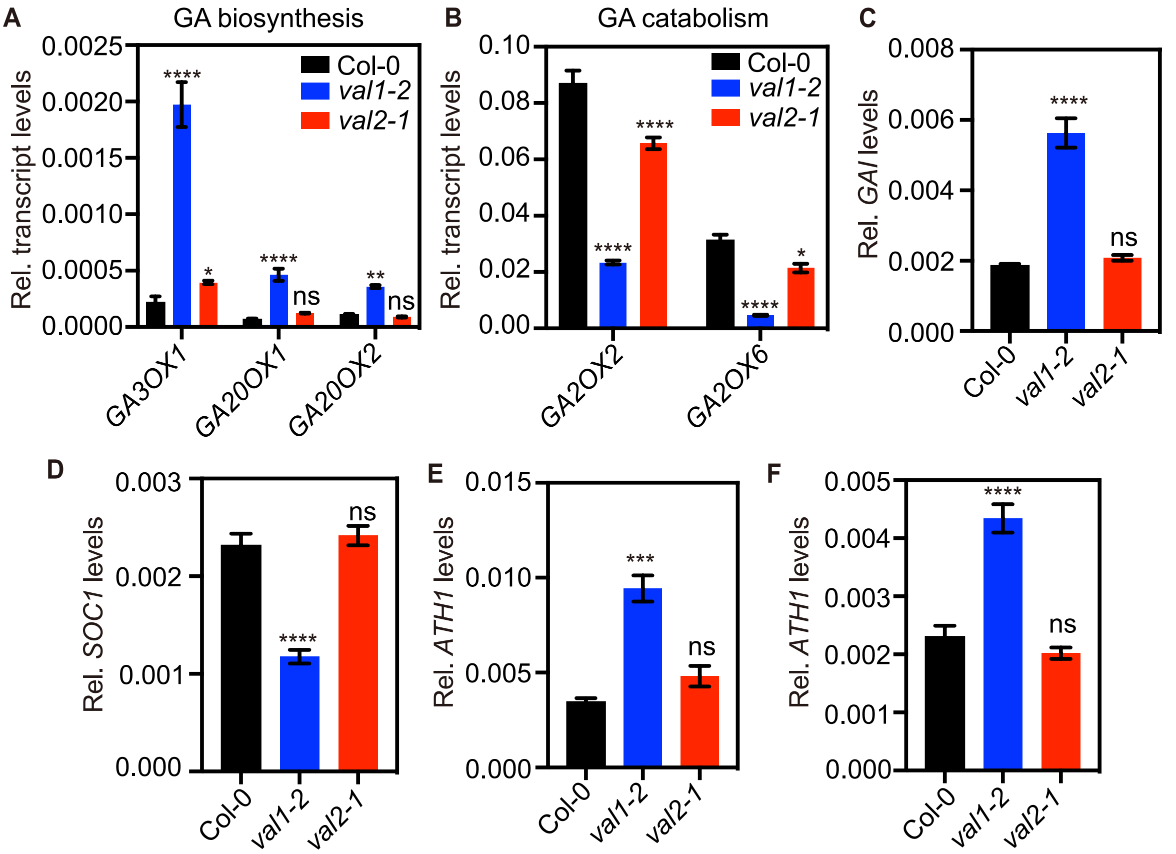

Supplementary Figure S5 VAL1 regulates the transcription level of flowering and gibberellin (GA) pathway genes. (A-E) Relative (Rel.) transcript levels of three GA biosynthesis genes (A; n=9), two GA catabolism genes (B; n=9), the DELLA gene *GAI* (C; n=9), the floral integrator gene *SOC1* (D; n=9), the homeobox gene *ATH1* (E, n=3) expressed in the SAM of 6-week-old Col-0 (WT), *val1* and *val2* plants grown under short days (SDs).
(F) Relative (Rel.) transcript levels of *ATH1* in 2-week-old seedlings grown in long days (LDs). Transcript abundance was measured by RT-qPCR. Values are means ±SEM. *ACTIN2* was used as normalization control. Different stars indicate significant differences as determined using Ordinary two-way ANOVA Multiple comparisons for GA-related genes and floral integrator genes, and Ordinary one-way ANOVA Multiple comparisons for *ATH1* mRNA (**P* < 0.05; ***P* < 0.01; *****P*<0.0001; ns, not significant).

Figure S6

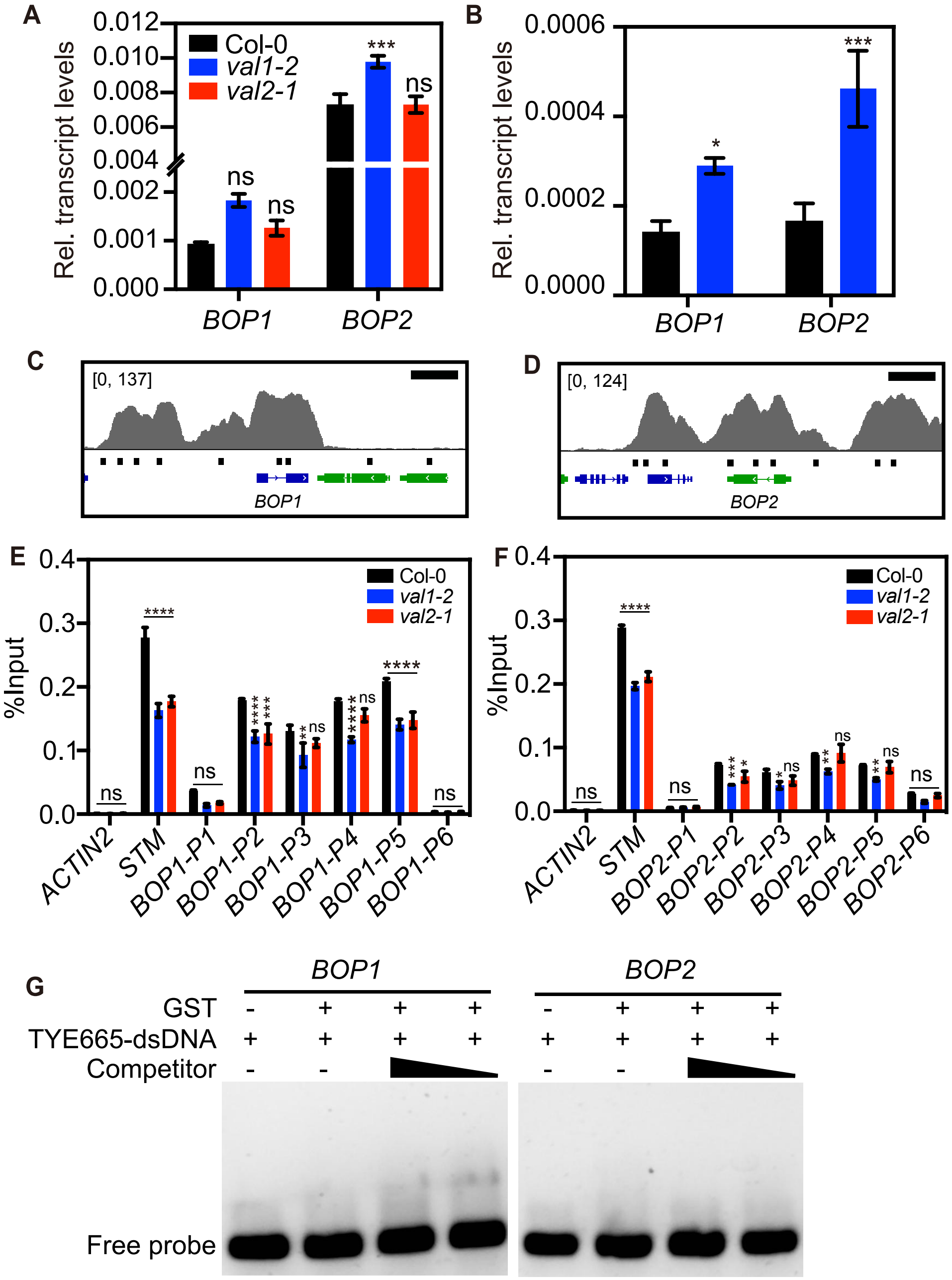

Supplementary Figure S6 VAL1 induces repression of *BOP1* and *BOP2* via the PRC2 pathway.
(A, B) Relative (Rel.) transcript level of *BOP1* and *BOP2* were detected in 2-week-old seedlings of Col-0 (WT), *val1* and *val2* plants (A) and in 11-day-old seedlings of WT and *val1* plants (B) at long day (LD) conditions. *ACTIN2* and *UBQ10* were used as normalization control. Different stars indicate significant differences as determined using Ordinary two-way ANOVA Multiple comparisons (*P < 0.05, ***P<0.001, ns, not significant).
(C, D) H3K27me3 ChIP-seq coverage tracks over *BOP1* (C) and *BOP2* (D) genomic loci. Data were extracted from publicly available ChIP-seq data (Yuan et al, 2021). Black boxes below the tracks and above the gene structures indicate the distribution of RY elements in the *BOP1* and *BOP2* genomics regions. Scale bar, 2kb.
(E, F) ChIP-qPCR analysis of H3K27me3 enrichment at *BOP1* (E) and *BOP2* (F) loci in 2-week-old WT, *val1* and *val2* plants grown under LDs. *SHOOT MERISTEMLESS* (*STM*) and *ACTIN2* were used as positive and negative controls of H3K27me3 enrichment, respectively. Values are means ±SEM of the three technical replicates in one qPCR experiment. An independent biological replicate of this H3K27me3 ChIP experiment is presented in Figure 5G-H. Different stars indicate significant differences as determined using Ordinary two-way ANOVA Multiple comparisons (*P < 0.05, **P < 0.01, ****P<0.0001, ns, not significant).
(G) EMSA testing GST (negative control) binding to the RY motif of *BOP1* (left) and *BOP2* (right) with decreasing concentration of competitor. Complementary to Figure 5F.
